# Anisotropic magnetoresistance: materials, models and applications

**DOI:** 10.1098/rsos.230564

**Published:** 2023-10-18

**Authors:** Philipp Ritzinger, Karel Výborný

**Affiliations:** ^1^ FZU—Institute of Physics, Academy of Sciences of the Czech Republic, Cukrovarnická 10, Praha 6 16253, Czech Republic; ^2^ MFF—Faculty of Mathematics and Physics, Charles University, Ke Karlovu 5, Praha 2 12000, Czech Republic

**Keywords:** resistance, anisotropic magnetoresistance, ferromagnets, antiferromagnets, transition metals, sensors

## Abstract

Resistance of certain (conductive and otherwise isotropic) ferromagnets turns out to exhibit anisotropy with respect to the direction of magnetization: $R_{∥}$ for magnetization parallel to the electric current direction is different from *R*_⊥_ for magnetization perpendicular to the electric current direction. In this review, this century-old phenomenon is reviewed both from the perspective of materials and physical mechanisms involved. More recently, this effect has also been identified and studied in antiferromagnets. To date, sensors based on the anisotropic magnetoresistance (AMR) effect are widely used in different fields, such as the automotive industry, aerospace or in biomedical imaging.

## 1. Introduction

The electric resistance *R* of a conductor depends on the state of its magnetic order; for example, in ferromagnetic metals at saturation, it depends on the direction of magnetization M. Experimentally, control of external magnetic field B allows to change M and this suggests the name magnetoresistance. The reader should not be misled into thinking that any dependence *R*(*B*) is confined to magnetically ordered materials though. Magnetoresistances (MRs) encompass a wide range of phenomena and in this review, we only focus on situations where the *anisotropy* of *R* is caused by magnetic order. By large part, we will discuss ferromagnets (FMs) where such anisotropic magnetoresistance (AMR) has been explored extensively but only a few reviews exist and the most popular McGuire & Potter [1] article is now almost half a century old (newer reviews [2], or section 15.3.3 in [3] have garnered relatively little attention). More modern developments in the field will also be discussed, whereas there seems to be a shift of focus from FMs to materials with more complex magnetic order (of which antiferromagnets are of particular interest) and here, even an elementary consensus on terminology is still to be reached.

After this introductory section, we turn our attention to approaches to model and thus understand the AMR (§2) and then, to materials where AMR has been explored (§3). AMR applications are listed in §4.

### 1.1. Basic observations

The basic approach to quantify AMR in a given ferromagnetic material is to compare resistance for magnetization parallel and perpendicular to current direction relative [4] to their suitably chosen average *R*_0_

1.1 $$\text{AMR}=\frac{R_{∥}-R_{⊥}}{R_{0}}.$$

Depending on the context, the most obvious choice $R_{0}=(R_{∥}+R_{⊥})/2$ may be replaced by another weighted sum [1], but since AMR is typically of the order of per cent, this is usually of little consequence. AMR in most metals is positive and it depends on temperature: it vanishes when magnetic order is lost upon heating.

**Figure 1.**
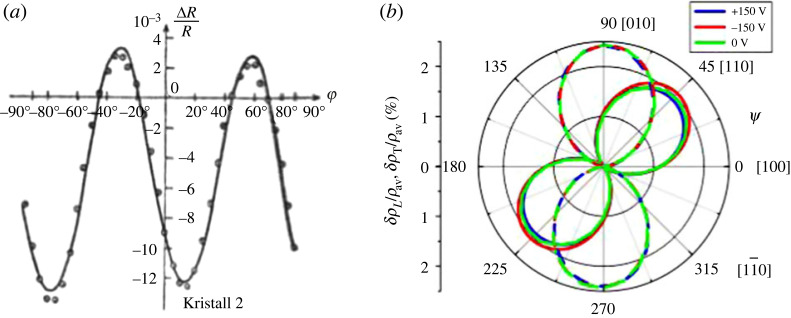
Two basic examples of AMR measurement. (*a*) Döring's measurements on nickel crystals and (*b*) longitudinal and transversal AMR measured on (Ga,Mn)As thin films. Reproduced from (*a*) Döring [5] and (*b*) De Ranieri *et al.* [6].

A more careful analysis of AMR requires the consideration of full resistivity tensor *ρ*_*ij*_. In a single crystal (of sufficiently low symmetry), anisotropies appear even in the absence of magnetic order and the AMR must not be confused with these ‘fundamental anisotropies’. Even cubic systems can, however, exhibit non-zero off-diagonal components of *ρ*_*ij*_ under non-zero magnetization (see §1.3) and to this end, angular dependence of *ρ* should be considered; the most common observation is

1.2 $$\frac{\rho_{xx}}{\rho_{0}}=1+C_{I}\cos2\varphi ,$$

where *C*_*I*_ is sometimes called non-crystalline AMR because it survives (as opposed to the more complex angular dependences discussed in §2.1 for example) even in polycrystalline systems. Clearly, the AMR as defined in equation (1.1) is just twice *C*_*I*_ when no crystalline AMR is present. A basic example of such an angle-resolved AMR measurement can be found in figure 1 for nickel and (Ga,Mn)As, respectively.

Angle φ in equation (1.2) refers, in the thin-film geometry (figure 3), to magnetization direction $\hat{m}$ rather than to the magnetic field B (associated with angle *α* in that sketch). The former is controlled by B and the link may be provided by Stoner–Wohlfarth model discussed in §§1.4 and 1.5. This approach is generally valid for systems with a single spin axis (SSA), thus FMs and collinear antiferromagnets. For other systems, such as non-collinear systems, the situation is more complicated and will be discussed in §4.2.3.

### 1.2. History and more features

*Discovery.* Transition metals (TMs) were the first materials where AMR was discovered: in 1857, William Thomson measured [7] in Fe and Ni what we would call non-crystalline AMR nowadays. The discovery in the third elemental room temperature (RT) ferromagnet, Co, was made a little later [8]. While these measurements concerned polycrystalline samples, Döring in 1938 investigated [5] the AMR in Fe and Ni single crystals more thoroughly as a function of φ and also the angle of M respective to crystallographic directions. Apart from the non-crystalline AMR (equation (1.2)) terms dependent on crystal symmetry (crystalline AMR) were found. His phenomenological approach to describing the full AMR in single crystals is still frequently used in modern works [6,9–12] as discussed in §2.1.

*Intrinsic and extrinsic AMR.* Next to the possible classification into non-crystalline and crystalline AMR, we can also make the distinction between intrinsic and extrinsic contributions. In the simplest case of Drude formula,

1.3 $$\sigma_{0}=\frac{ne^{2}\tau }{m}=\omega_{\;p}^{2}\epsilon \tau \;\mathrm{and}\;\sigma (\omega )=\frac{\sigma_{0}}{1-i\omega \tau },$$

the extrinsic (thus scattering-dependent) effects enter through the dependence of relaxation time *τ* on the magnetization direction while the intrinsic contribution to AMR amounts to such a dependence of the plasma frequency *ω*_*p*_. Examples of the former mechanism can be captured by effective models described in §2.2.1 and the prime example is s-d-scattering, thus, that delocalized conduction electrons (4s) are scattering into localized 3d states via magnetic impurities (this zero-temperature mechanism [1] is of course not the only type of scattering that can lead to anisotropic transport). Intrinsic AMR receives more attention in recent years [13,14], since investigated materials are generally more complicated and band structure calculation has become more precise, allowing for a more thorough distinction. On a theoretical side, AMR can be calculated from the band structure (intrinsic contribution) and is then compared with experimental results. If there happens to be a significant difference, this can be attributed to scattering (extrinsic contribution). Experimentally, the usage of AC-voltage can be used to distinguish [15] the intrinsic and extrinsic contributions to *σ*(*ω*) in equation (1.3), since the intrinsic contribution leads to frequency-independent terms in AMR, while the extrinsic contribution scales with 1/*ω* (see §4.2.1 for details).

*Negative AMR.* In most common metals, AMR as defined by equation (1.1) is positive; this is fairly demonstrated by table 1, which also shows also one of the early examples of systems where AMR is negative (cobalt with traces of iridium). The first materials where negative AMR was found were, nevertheless, much more common alloys of transition metals with aluminium [25]. The belief that negative AMR is an exception established itself in the next couple of decades, which may be fuelled by the fact that major theories of AMR were developed on simple transition metals showing positive AMR under normal circumstances.

**Table 1.** Examples of AMR values for three groups of TM-based systems: pure room temperature (RT)-FM metals (i.e. Fe, Co, Ni); the basic TM with TM-impurity; and alloys of the three basic TM. More examples of Ni-alloys with other TM impurities can be found in table 1 of Jaoul *et al.* [16] and more examples of alloys with Ir as an impurity are listed in table 1 of McGuire *et al.* [17]. AMR for other concentrations of Pd in Co–Pd are listed in table 1 of Jen [18], where the given composition Co_45_Pd_55_ shows the maximum value. A broader listing of the nickel-based alloys FeNi, CoNi and (CoNi)Fe is found in [19] and of the iron-based alloys NiFe, FeCr, FeV and FeCo in fig. 1*b* and fig. 2 of Berger *et al.* [20]. AMR values for NiFeCr with higher concentrations of Cr are listed in table 1 of Chakraborty & Majumdar [21]. See §3.1 for discussion of the transition metals.

material	AMR percentage	remarks
Fe	0.2–1.5	RT [22] to low temperature (LT) [23]
Ni	1.8–3.15	see table I in [4] and also fig. 1*a*
Co	0.3–3.5	from El-Tahawy *et al.* [24]; fig. 1 in [25]
Ni with Pd	2	*T* = 4.2 K; impurity without VBS [16]
Ni with Zn	6.5	low temperature; impurity without VBS [16]
Ni with Cr	−0.28	*T* = 4.2 K; impurity with VBS [16]
Co with 3% Ir	−2.56	RT; [17]
Co_45_Pd_55_	7.96	*T* = 4 K; [18]
Ni_80_Fe_20_ (permalloy)	16–25	LT and RT [20]; *T* = 10 K [19]
(Ni_100−*x*_Co_*x*_)_86_Fe_14_	in excess of 50	for x≈20% [19], see discussion in text
Ni_77_Fe_22_Cr_2_	0.76	*T* = 4.2 K; [21]

**Figure 2.**
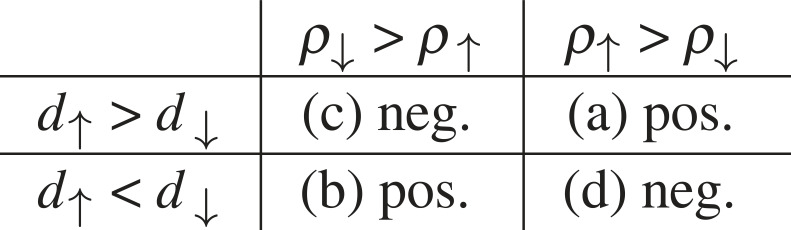
Sign of AMR explained in the context of *sd*-model. Examples: (a) bcc Fe, (b) fcc Co or Ni, (c) half-metallic FMs such as Co_2_MnAl_1−*x*_Si_*x*_ and (d) Fe_4_N. Inspired heavily by fig. 4 of Kokado *et al*. [27].

One of the main approaches to microscopically understanding the AMR, so-called *sd*-model which is explained in §2.2.1, allows to understand the AMR sign (in some materials) using the following simplified picture based on Mott’s two-current model [26], which operates with two spin channels and their resistivities *ρ*_↑,↓_. We will follow explanations by Kokado *et al.* [27], where the density of states (DOS) at Fermi level *E*_*F*_ in the majority/minority *d*-bands is *d*_↑_/*d*_↓_. Figure 2 shows the relation between the AMR sign and the dominant *s* → *d* scattering process. Namely, the *s*_↑_ → *d*_↓_ scattering or *s*_↓_ → *d*_↑_ scattering is responsible for positive AMR, while the *s*_↑_ → *d*_↑_ scattering or *s*_↓_ → *d*_↓_ scattering cause negative AMR. The key parameter is thus *α* = *ρ*_↓_/*ρ*_↑_ and a detailed discussion [27] serves as a useful guideline for the AMR sign across the whole material class of transition metals. Validity of this guideline is limited, however, by the range of applicability of the *sd*-model: other material classes, such as dilute magnetic semiconductors (DMSs) discussed in §3.2, follow different patterns [28,29].

In the context of this theory [27], negative AMR is sometimes promoted to be a possible sign of half-metallicity [30–32], which has to be taken with caution: first, the sign of AMR as defined by equation (1.1) may depend on the current direction with respect to the crystal (in which case it makes better sense to analyse AMR in terms of its non-crystalline and crystalline components, see §2.1) and this clearly cannot mean that the system would be half-metal in one case and normal metal in the other case. An example of a material which is clearly *not* a half-metal is the 30:70 alloy of iron and cobalt [33] (sign change of AMR can be seen in fig. 2 of that reference where B and M are nearly parallel). Also, temperature variation can cause similar changes (e.g. in Mn_4_N [34]). Second, even in predominantly negative signed Co-based Heusler alloys, positive AMR was reported by e.g. variations of the stoichiometry [32] or the annealing temperature [35] (see §3.4). The changes of sign in all of these materials were explained successfully within the framework of the aforementioned majority/minority scattering by Kokado and Tsunoda.

Still, it holds that the half-metallic DOS induces a negative sign of AMR. The backward conclusion (negative sign implies half-metallicity [33]) is not generally true. Other systems where AMR can be negative will be discussed later in this review: certain antiferromagnets, manganites and two-dimensional electron gases to name a few.

### 1.3. Anisotropic magnetoresistance and the more fancy effects

We first wish to elucidate the relationship of AMR to off-diagonal component of the resistivity tensor

**Figure 3.**
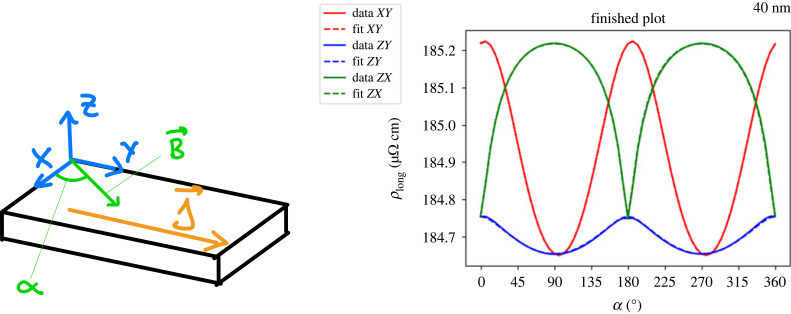
Example of Stoner–Wohlfarth analysis in AMR data of a Co_2_MnGa thin-film sample. Alongside the SW1 model and a basic non-crystalline AMR, also higher-order crystalline AMR terms are taken into account (see §2.1). There is an excellent agreement between data and fit. The magnetic field was rotated in three different rotation planes denoted as *XY*, *ZY* and *ZX*, where $Z=\hat{n}∥[001]$, $Y=\hat{j}∥[110]$ and *X* = *Y* × *Z*. The rotation in the *XY*-plane begins at the *X*-axis and in the other plane at the *Z*-axis. Reproduced from Ritzinger [37].

1.4 $$\frac{\rho_{xy}}{\rho_{0}}=C_{I}\sin2\varphi ,$$

in the simplest case, which is often called the planar Hall effect (PHE) even if transversal AMR [6] seems a more appropriate name. Assume a planar system with magnetization $\hat{m}∥\hat{x}$ which would be otherwise isotropic (in other words, $\hat{m}$ provides the only source of symmetry breaking). Let us denote the two non-zero components *ρ*_*xx*_ and *ρ*_*yy*_ by $\rho_{∥}$ and *ρ*_⊥_, respectively. Now consider a rotation of $\hat{m}$ to $R_{\phi }\hat{m}$: in a polycrystal, this would be equivalent to leaving m unchanged and rotating the resistivity tensor instead,

1.5 $$R_{\phi }\left(\begin{array}{cc} \rho_{∥} & 0\\ 0 & \rho_{⊥} \end{array}\right)R_{\phi }^{T}=\left(\begin{array}{cc} \rho_{0}+\frac{1}{2}\Delta \rho \cos2\phi & \frac{1}{2}\Delta \rho \sin2\phi \\ \frac{1}{2}\Delta \rho \sin2\phi & \rho_{0}-\frac{1}{2}\Delta \rho \cos2\phi \end{array}\right),$$

where $\Delta \rho =\rho_{∥}-\rho_{⊥}$ and $R_{\phi }$ is an orthogonal matrix. The off-diagonal elements can be identified with equation (1.4) and hence our terminological preference (transversal AMR rather than PHE). We point out, however, that ‘transverse AMR’ is sometimes used [36] to describe the experimental configuration where magnetization rotates in the plane perpendicular to the current direction (green curve shown in figure 3); in equations (1.2) and (1.4), this corresponds to constant *ϕ* = *π*/2 and one would then naively expect no variation of resistance. We explain in §2.1 that *crystalline AMR* is responsible for any signal measured in this set-up.

AMR belongs to a wider family of transport phenomena in magnetically ordered materials and in the following we mention several further examples of its members. They are all bound by Onsager reciprocity relations, for resistivity tensor they read

1.6 $$\rho_{ij}(M,B)=\rho_{\;ji}(-M,-B).$$

We observe that for *ρ*_*xy*_ , this relation can be fulfilled either by equation (1.4) in the transverse AMR (a symmetric tensor component *ρ*_*xy*_ = *ρ*_*yx*_ which is even in magnetization) or by the anomalous Hall effect (AHE) with *ρ*_*xy*_ = −*ρ*_*yx*_ odd in magnetization. More complex cases are discussed in [38]. Next, there are thermoelectric counterparts of these effects, the anomalous Nernst effect (to AHE) and the anisotropic magnetothermopower (AMTP) discussed in §4.2.3. Spin conductivity instead of charge conductivity can also be studied (e.g. SHE instead of AHE) and both effects are closely related [39], e.g. in permalloy, AHE scales with the spin Hall effect (SHE) in proportion to the spin-polarization. Finally, we wish to mention transport in ballistic rather than diffusive regime: tunnelling AMR (TAMR) and ballistic AMR discussed in §4.2.4.

### 1.4. What anisotropic magnetoresistance is and what it is not

MR may refer to any phenomenon [40] where *R*(*B*) is not constant and, as such, they are not limited in scope to magnetically ordered materials. Orbital effects leading to MR imprint the anisotropy of crystal to *R*(*B*), as recently nicely reviewed by Zhang *et al*. [41], and ensuing anisotropic orbital (or ordinary) MR related simply to the Lorentz force acting on electrons [42] is *not* the subject of the present review; neither is the MR of surface states in topological insulators that also exhibits anisotropy [43] and other magnetoresistive effects in non-magnetic systems which are not isotropic. Only occasionally these effects are kept separate from AMR, laudable exception being eq. (1) in [44] for example, whereas disentangling the two contributions is not trivial. To this end, a meaningful first step is to measure *R*(*B*) curves for two directions of B (parallel and perpendicular to current) and verify if the two curves remain parallel beyond certain value of *B* which can reasonably be associated with the saturation of magnetic order.

On the other hand, the AMR appears under different names in literature: spontaneous magnetoresistance anisotropy (SMA) [45], spontaneous resistivity anisotropy (SRA) [46,47], ferromagnetic anisotropy of resistivity (FAR) [21] or magneto-resistivity anisotropy [48]. Also, longitudinal MR and transversal MR are sometimes discussed separately [24], whereas their difference in a high magnetic field is the actual AMR. On some occasions, the term AMR or anisotropic MR is used, when the MR ratio is plotted for different field directions [49,50]. In that case, it can be that the AMR ratio is not quantitatively calculated as in equation (1.1), but the discussion is rather restricted to the mere fact that the MR is different for different field directions, thus implying AMR. Ideally, we are interested in magnetically ordered materials at saturation.

*Misconception with MCA.* A frequent trouble is the confusion of AMR and magnetocrystalline anisotropy (MCA). Whenever there is a deviation from the classical twofold dependence $\Delta \rho \propto \cos^{2}$ (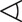 (H,J)) (where H and J is the current density) it is not *per se* clear whether they stem from MCA or are AMR terms. MCA can lead to higher-order symmetries on the AMR signal; however, these terms might also originate from AMR due to crystalline symmetry (so-called *crystalline AMR* or *single-crystal AMR* (SCAMR)). The frequent conclusion, the higher-order terms stemming from MCA is only unequivocally true in polycrystalline materials. In single crystals a careful distinction of these MCA and AMR is always a must (e.g. by determining the value of MCA in a different experiment and accounting for it). Furthermore, it should be kept in mind that crystalline AMR and MCA do not have the same effect: while both are dependent on the band structure, a key ingredient of any (extrinsic) AMR is scattering, which does not play a role in MCA. The intrinsic AMR depends on the anisotropy of the Fermi velocities, which is not necessarily linked to the exchange energy causing the MCA into existence. The concept of MCA is further elaborated in §1.5 and the crystalline AMR is derived and explained in detail in §3.1. An illustration of the difference between AMR and MCA can be seen in fig. 4*d*–*g* of Alagoz *et al.* [48], where the AMR and MCA show much different temperature dependences.

### 1.5. Magnetization control

AMR in its essence is a spontaneous effect. This can be illustrated by comparing the zero-field extrapolation of the MRs $\rho_{∥}(B)$ (also longitudinal MR, or LMR) and *ρ*_⊥_(*B*) (also transversal MR, or TMR), which allow defining zero-field AMR (zf-AMR) [44]. In some situations, it is even possible to prepare the system in two states, stable at *B* = 0, with different magnetic configurations. Since in applications such as readout heads or sensors, the applied magnetic field is a key ingredient, the discussion about whether AMR can finally be considered a spontaneous effect is of little relevance for this review, and in practice, it is usually the applied magnetic field that steers the magnetic moments. In other words, it is desirable to determine the magnetic state depending on B; alternative ways of manipulation of magnetic moments will be discussed in §4.2.3. First, we assume that we are looking at a single-domain state (effects related to a non-trivial domain structure tend to be more severe in antiferromagnets [51]); next, we focus only on classical magnetism. Under these assumptions, we are left with inter-sublattice exchange coupling (if there is more than just one magnetic sublattice) and magnetic anisotropy.

A convenient framework in FMs is the time-proven Stoner–Wohlfarth model [52] (henceforth referred to as the SW1 model) which writes as [53],

1.7 $$\frac{E}{MV}=-B\hat{b}\cdot \hat{m}+B_{a}{\left(\hat{m}\cdot \hat{a}\right)}^{2},$$

which yields the local energy minimum for magnetization depending on history and two parameters: B=|B| and *B*_*a*_ (magnetic anisotropy). Among others, SW1 models are widely used in the analysis of resistivity data. An example can be found in figure 3, where next to basic non-crystalline AMR, an SW1 model and higher-order crystalline AMR components were taken into account (see §2.1 for the latter); the latter becomes manifest in (a) the different amplitudes of the blue and red curves or (b) a non-constant signal plotted as the green curve. As for (b), magnetization remains always perpendicular to current, φ = *π*/2, and if equation (1.2) were the complete description of AMR in this case, *ρ*_*yy*_ should remain constant. By including *crystalline AMR* terms into equation (1.2) as discussed later (see equation (2.4)), the observed behaviour both for (b) and (a) can be well understood. The same type of description (based on SW1, see figure 8) was used by Limmer *et al.* [10,54] for (Ga,Mn)As.

As soon as there is more than one magnetic sublattice (MSL), the situation becomes less straightforward [55]. It is possible to generalize the previous approach to antiferromagnets with two MSLs: such SW2 model reads

1.8 $$\frac{E}{MV}=B_{e}{\hat{m}}_{1}\cdot {\hat{m}}_{2}-B\hat{b}\cdot ({\hat{m}}_{1}+{\hat{m}}_{2})+B_{a}[{\left({\hat{m}}_{1}\cdot \hat{a}\right)}^{2}+{\left({\hat{m}}_{2}\cdot \hat{a}\right)}^{2}],$$

and a new parameter has been introduced: the inter-sublattice exchange coupling *B*_*e*_. The basic mode of operation of SW2 [56] is that the Néel vector $L={\hat{m}}_{1}-{\hat{m}}_{2}$ is perpendicular to B, which always (for |*B*| > 0) corresponds to energy minimum in equation (1.8) once *B*_*a*_ = 0. In this way, L can be effectively controlled by B and for finite *B*_*a*_, the same applies beyond spin-flop field $\propto \sqrt{B_{a}B_{e}}$.

This concept can be extended to more complicated systems, and starting with SW3, non-collinear magnetic order has to be considered. Recently, Mn_3_X materials (where X can be Ge or Sn, for example) attracted significant attention and Liu & Balents [57] discuss a model where beyond adding a third MSL to equation (1.8) also Dzyaloshinskii–Moriya interaction is included. The geometry of Kagome lattice (figure 16) introduces frustration, and relationships between B and ${\hat{m}}_{1,2,3}$ are in general difficult to describe in simple terms. All such macrospin models the reality of multi-domain states [58] and modelling of these involves assumptions about population of individual energy minima of equation (1.8) as, for example, in [51].

## 2. Modelling

In this section, the different modelling approaches are presented. We will start in §2.1 by introducing potent phenomenological models, which allow us to effectively analyse the even most complex AMR data. Due to their phenomenological nature, however, they cannot give insight into the possible origins of individual terms in expansions such as equation (2.4). While more involved, microscopical models reviewed in §2.2 make such a deeper understanding possible.

### 2.1. Phenomenological models

We define the magnetic field direction to be $\hat{h}=H/H$ and the magnetization direction to be $\hat{m}=M/M$. Please keep in mind that the AMR depends on $\hat{m}$ and not on $\hat{h}$—the rotation of the magnetic field is simply used to control the rotation of the magnetization. The dependence of $\hat{m}$ on $\hat{h}$ was discussed in the previous section and the confusion of MCA with AMR in §1.4. Speaking in somewhat loose terms, it holds that $\mathrm{AMR}\propto \rho (\hat{m})\neq \rho (\hat{h})$.

The simplest possible way to describe the AMR presents itself as equation (1.2): $\Delta \rho (\hat{m})\propto \cos(2\varphi )$, where φ is the angle between $\hat{m}$ and current direction $\hat{j}=J/J$. In a single crystal, this simple picture does not hold anymore, but instead, the AMR can have more complex contributions depending on the crystalline symmetry. In the following section, we will present a simple yet extremely powerful phenomenological model to describe (however, *not* explain) even complex AMR data, which was originally developed by Döring in 1938 [5] and since then used many times again [6,9–12,54].

*The model.* To begin with, we assume that we do not know the correct analytical expression of the resistivity *ρ* and that *ρ* depends only on the direction of the magnetization $\hat{m}$. Furthermore, there can be higher-order dependencies on $\hat{m}$. Thus, we express *ρ* as a power series of $\hat{m}$:

2.1 $$\rho_{\mathrm{ij}}(\hat{m})=\rho_{\mathrm{ij}}^{\left(0\right)}+\rho_{\mathrm{ijk}}^{\left(1\right)}m_{k}+\rho_{\mathrm{ijkl}}^{\left(2\right)}m_{k}m_{l}+\rho_{\mathrm{ijklm}}^{\left(3\right)}m_{k}m_{l}m_{m}+\rho_{\mathrm{ijklmn}}^{\left(4\right)}m_{k}m_{l}m_{m}m_{n}+\cdots ,$$

where $\rho_{\mathrm{ij}}^{\left(0\right)}$, $\rho_{\mathrm{ijk}}^{\left(1\right)}$, $\rho_{\mathrm{ijkl}}^{\left(2\right)}$, $\rho_{\mathrm{ijklm}}^{\left(3\right)}$ and $\rho_{\mathrm{ijklmn}}^{\left(4\right)}$ are the expansion coefficients and $m_{k},m_{l},m_{m},m_{n}\in \{m_{\left[100\right]},m_{\left[010\right]},m_{\left[001\right]}\}$ are the cartesian components of $\hat{m}$.

The number of independent parameters is reduced by using the following four strategies: (i) commutation *m*_k_ *m*_l_ = *m*_l_ *m*_k_ for all *m*_k_ and *m*_l_, (ii) the identity $m^{2}=\sum_{k}m_{k}^{2}=1$, (iii) the Onsager relation [9], $\rho_{\mathrm{ij}}(\hat{m})=\rho_{\mathrm{ji}}(-\hat{m})$, and (iv) Neumann’s principle: the resistivity tensor, as well as its expansion coefficients, must reflect the crystal symmetry [59]. There are several ways to account for the symmetry, e.g. by using generator matrices of the crystal symmetries as shown in [9,10,54].

**Figure 4.**
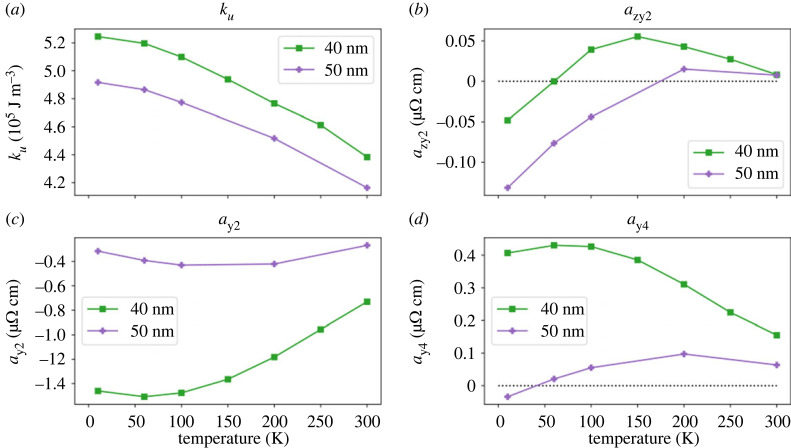
Temperature evolution of the phenomenological parameters obtained by the fit to AMR data of two Co_2_MnGa thin-film samples. (*a*) Uniaxial magnetic anisotropy from the SW1 model and (*b*–*d*) part of the parameters for AMR in tetragonal symmetry similar to those defined in equation (2.2). Reproduced from Ritzinger *et al.* [12].

For a more detailed treatment, it can be wise to consider the previously mentioned publications, especially the treatment in [9]. Next, we explain how the great number of coefficients appearing in equation (2.1) can be reduced to a small set of key parameters such as those shown in figure 4 for a specific tetragonal system.

Please note that this approach yields an expression for the resistivity tensor *ρ*_*ij*_ differing depending on the crystal symmetry. The tensor in cubic symmetry can (among others) be found in eq. (4) of Limmer *et al.* [54] and in tetragonal symmetry in eqs. (4) and (5) of Limmer *et al.* [54]. The resulting tensor depends generally on the components *m*_k_ of $\hat{m}$ and also on coefficients *A*, *B*, …, which are unknown in the general case and are sample-dependent.

The longitudinal resistivity *ρ* is obtained by applying Ohm’s Law: $\rho =\hat{j}\rho_{ij}\hat{j}$. The coefficients of the resistivity do change depending on the crystal symmetry and the current direction. As an example, *ρ* in cubic symmetry with $\hat{j}∥\text{[100] }\equiv j_{x}$ writes as

2.2 $$\begin{array}{rl} \rho & =\rho_{0}+a_{x2}\cdot m_{x}^{2}+a_{x4}\cdot m_{x}^{4}+a_{\mathrm{zy}2}\cdot m_{z}^{2}\cdot m_{y}^{2}\\ & =\rho_{0}+a_{x2}\cos^{2}\phi \sin^{2}\theta +a_{x4}\cos^{4}\phi \sin^{4}\theta +a_{\mathrm{zy}2}\sin^{2}\phi \sin^{2}\theta \cos^{2}\theta , \end{array}$$

where the *a*_x2_, *a*_x4_, *a*_zy2_ are effective sample-dependent coefficients, which are linked to the original set of coefficients *A*, *B*, …, and in the second step a parametrization of $\hat{m}$ in polar coordinates $\hat{m}=(\cos(\phi )\sin(\theta ),\sin(\phi )\sin(\theta ),\cos(\theta ))$ was applied. The calculations are lengthy and can be found elsewhere, alongside expressions for the longitudinal resistivity for current along [110]  or the resistivity tensor for tetragonal crystal symmetry [9,10,12,54]. Expressions for other symmetries in literature are not known to us. These phenomenological coefficients are temperature dependent (as an example, data from two Co_2_MnGa thin-film samples with tetragonal symmetry can be found in figure 4*b*–*d*) and they are only sometimes [28] monotonous. The same approach can be applied to describe transversal resistivity *ρ*_trans_. In systems of cubic symmetry with j∥[100], it writes as

2.3 $$\begin{array}{rl} \rho_{\mathrm{trans}} & =a_{z1}\cdot m_{z}+a_{\mathrm{xy}}\cdot m_{x}\cdot m_{y}+a_{z3}\cdot m_{z}^{3}+a_{\mathrm{xyz}2}\cdot m_{x}m_{y}m_{z}^{2}\\ & =a_{z1}\cos\theta +a_{\mathrm{xy}}\cos\phi \sin\phi \sin^{2}\theta +a_{z3}\cos^{3}\theta +a_{\mathrm{xyz}2}\sin\phi \cos\phi \sin^{2}\theta \cos^{2}\theta , \end{array}$$

and here, contributions odd and even in $\hat{m}$ are mixed. Besides the Hall effects (e.g. AHE *a*_z1_ and its anisotropic part *a*_z3_), there are also even terms: the transversal AMR. In polycrystalline systems, they are reduced to *a*_xy_ (corresponding to the non-crystalline term *C*_*I*_), while in single crystals higher-order contributions can occur in both longitudinal and transversal AMR, which means that considering transversal AMR could yield additional information about the system [60]. Equation (2.2) is only one possible way of writing things down. For example, Döring [5] expresses the resistivity in terms of direction cosines of the magnetization *α*_*i*_ and of the current *β*_*i*_. Another way of describing the AMR (assuming in-plane configuration as in figure 3) is given by [6],

2.4 $$\frac{\Delta \rho_{\mathrm{long}}}{\rho_{\mathrm{av}}}=\underset{\mathrm{non}-\mathrm{crystalline}}{\underbrace{C_{I}\cdot \cos2\varphi }}+\overset{\mathrm{uniaxial}\;\mathrm{crystalline}}{\overbrace{C_{U}\cdot \cos2\psi }}+\underset{\mathrm{cubic}\;\mathrm{crystalline}}{\underbrace{C_{C}\cdot \cos4\psi }}+\overset{\mathrm{mixed}\;\mathrm{non}-\mathrm{crystalline}/\mathrm{crystalline}}{\overbrace{C_{IC}\cdot \cos(4\psi -2\varphi )}},$$

where φ is the angle between $\hat{m}$ and $\hat{j}$, and *ψ* is the angle between $\hat{m}$ and a certain, fixed crystallographic direction in the plane of rotation; note that the last term can also be written as cos (2φ + 2*θ*). Equations (2.4) and (2.2) are consistent, as shown in [37]. However, equation (2.4) is only a two-dimensional equation ($\hat{m}$ rotated in the plane of the surface), while equation (2.2) is a three-dimensional equation (AMR can be described for arbitrary $\hat{m}$ on a spherical surface via *ϕ* and *θ*). The correspondence between equations (2.4) and (1.2) (relevant for single-crystalline systems and polycrystals) has its counterpart also with *ρ*_*xy*_: equation (1.4) in polycrystals corresponds to [28] Δ*ρ*_trans_/*ρ*_av_ = *C*_*I*_ sin 2φ − *C*_*I*,*C*_ sin (2*ψ* + 2*θ*) where φ = *ψ* − *θ*. Note that, for fixed *θ* = 0 or *π*, phenomenology of *ρ*_long_, *ρ*_trans_ seemingly reduces again to equations (1.2) and (1.4) but this time with unequal amplitudes *C*_*I*_ + *C*_*I*,*C*_ and *C*_*I*_ − *C*_*I*,*C*_. In other words, if *ρ*_long_ and *ρ*_trans_ of cos 2φ and sin 2φ form is measured where the amplitudes are different, it should be interpreted as crystalline AMR (or, according to terminology of Rushforth *et al.* [28], in terms of a mixed non-crystalline and crystalline term).

*Higher-order contributions* are due to crystal structure and thus only appear in single crystals or epitaxial materials with sufficient crystal quality. In polycrystalline materials, the AMR will be twofold (see equation (1.2)) as can be shown theoretically by averaging the resistivity tensor over all possible crystal orientations (see [9,10,54])—or even simpler, to set *ψ* ≡ 0 in equation (2.4), since crystalline directions do not have any meaning in the polycrystalline limit. In doing so one will recover equation (1.2). This emphasizes the usage of the terms non-crystalline (= independent of crystal structure and thus twofold) and crystalline AMR.

The origin of the crystalline AMR is still under active investigation. While many studies restrict themselves to the mere existence of e.g. a fourfold symmetry, the picture is more complex since the AMR consists of many contributions in various crystalline directions, as can be seen above and e.g. in [5,9–12,54]. While these studies are an accurate description of all the terms possibly existing in the AMR, microscopic studies are rare. For the case of a fourfold symmetry, the effective model of Kokado & Tsunoda [61] (and see the following section) showed that a tetragonal symmetry is needed for the fourfold term to appear. The appearance of fourfold terms in many technically cubic materials can be linked to tetragonal distortions induced to thin films by many substrates.

However, a study describing all the terms in equation (2.2) as well as a study for even higher-order terms, is still missing to date.

While relatively rare, higher-order crystalline terms have also been reported. In hexagonal crystal structures, sixfold AMR can emerge. This was reported for instance in antiferromagnetic MnTe [62], but also in two-dimensional electron gases on hexagonal [111] interfaces between transition-metal oxides as discussed further in §3.5. The highest symmetry reported is an eightfold symmetry measured in (Ga,Mn)As [6] and in (In,Fe)As [63]. In the latter case, it was explained by crystal field effects due to a zinc-blende structure.

### 2.2. Microscopic models

Regardless of the detailed structure of a microscopic model aiming to describe AMR in a particular material, two basic ingredients are needed: reasonably accurate knowledge of the electronic structure and that of momentum relaxation. On the level of equation (1.3), this was reduced to the plasma frequency which can be evaluated, see §2.2.1, from electron dispersion $E_{k}$

2.5 $$\omega_{\;p}^{2}=8\pi^{2}\hbar^{2}e^{2}\int \frac{d^{3}k}{{\left(2\pi \right)}^{2}}v_{x}^{2}\delta (E_{\vec{k}}-E_{F}),$$

and, regarding the momentum relaxation, to transport relaxation time, which can be accessed through the Fermi golden rule,

2.6 $$\frac{1}{\tau }=\frac{2\pi n_{imp}}{\hbar }\int \;dk^{\prime }\delta (E_{i}-E_{\;f})|M_{kk^{\prime }}{|}^{2}(1-\cos\theta_{kk^{\prime }}),$$

whereas we only consider scattering on static disorder (such as point defects in crystal with density *n*_*imp*_). In the following, we elaborate on two possible strategies to treat both these ingredients and even if equations (2.5) and (2.6) represent only *examples* of how electronic structure and scattering can be taken into account, any microscopic model of AMR must in some way consider them both. We proceed to explain effective models, whereas symbols appearing in the preceding equations will also be described. Our focus will be, in general, on systems with metallic conduction and other situations (such as hopping conduction or systems with bound magnetic polarons [64]) will not be discussed in this review.

#### 2.2.1. Effective models

Most transport phenomena depend on band structure solely in the vicinity of Fermi level *E*_*F*_.^1^ To that end, integral in equation (2.5) needs only limited knowledge of band structure (and Fermi velocity component *v*_*x*_); rather than using the band dispersion $E_{k}$ in the full energy range, its effective model can often be constructed, which is easier to handle and offers better insight, e.g. into how the magnetization direction and spin–orbit interaction influence the band anisotropy [66]. At this point, we remark that through such anisotropy, the plasma frequency (equation (2.5)) may become anisotropic: in a non-magnetic cubic crystal, for example, *ω*_*p*; *xx*_ = *ω*_*p*; *yy*_ but when magnetic order is present, $\hat{m}\|\hat{x}$ breaks this symmetry. For the definition of such anisotropic *ω*_*p*_ and its discussion related to intrinsic AMR, see [15].

Turning our attention to the scattering, we first remark that should the resistances in equation (1.1) be calculated as ∝1/*σ*_0_ of (equation (1.3)) for different directions of $\hat{m}$, whereas *τ* remains constant, the resulting AMR is independent of *τ*. In other words, while scattering had to be taken into account to obtain finite conductivity *σ*_0_, it does not influence the AMR. This is, however, only the simplest situation possible: in most cases, *τ* does indeed depend on the direction of $\hat{m}$ and this can either become manifest in the matrix elements *M*_*kk*′_ of the scattering operator (below, we give an explicit example) or the direction cosine in equation (2.6). The latter opens a pathway for the current direction to enter directly the calculation of scattering time: *τ* in the relaxation time approximation (RTA) [67] depends on k and the Boltzmann expression for conductivity [68] assigns the largest weight to τ(k) with k parallel to the current direction.

Such was the approach to understanding the AMR in elemental FMs (notably, nickel or iron) since the seminal work of Smit [69]. Two current model works [70] with the ratio *α* of resistivities in majority and minority spin channels (within what was later [16] called the Smit mechanism) and the difference $\Delta \rho =\rho_{∥}-\rho_{⊥}$ with respect to the direction of $\hat{m}$ can then be expressed as

2.7 $$\frac{\Delta \rho }{\rho }=\gamma (\alpha -1),$$

where *γ* ≈ 10^−2^ describes the competition of spin–orbit interaction and exchange interaction. For these simple cases, it holds that *α* > 1 (thus *ρ*_↓_(*T* = 0) > *ρ*_↑_(*T* = 0)), so that the AMR is always positive and the other cases are described in figure 2. It should be stressed that equation (2.7) provides only basic guidance to AMR, yet it is referenced occasionally up to nowadays [71] when interpreting experiments; we return to the discussion of *sd*-models applied to AMR in elemental metals and their alloys in §3.1 and proceed now to discuss the effective models in DMSs.

**Figure 5.**
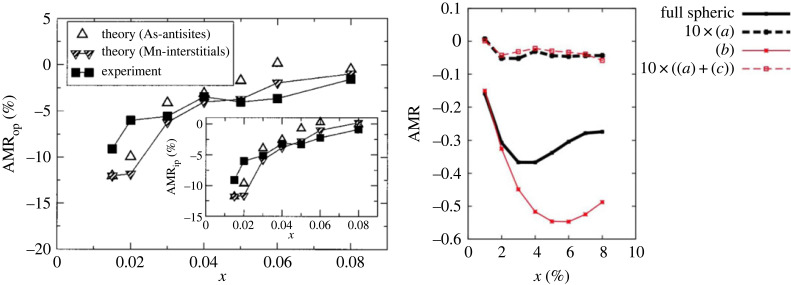
Left: measured AMR in the DMS (Ga,Mn)As with doping *x* varied [73]. Right: modelling allows to distinguish the intrinsic (*a*) and extrinsic (*b*,*c*) mechanisms of AMR; clearly, the extrinsic mechanism (*b*) as defined in [29] dominates. Reproduced from (left) [73] and (right) [29].

While the previously discussed *sd*-models [1] treat the band structure only on a rudimentary level, essentially *ω*_*p*_ in (1.3) is taken as coming from a single band and independent of $\hat{m}$, models of transport in DMS are more elaborate in this respect. The valence band $E_{k}$ can be obtained [72] from four- or six-band models (depending on the needed level of detail) and conductivity can be evaluated using the Boltzmann equation, see §3.2. It turns out [29] that the RTA with a constant (magnetization-direction independent) *τ* leads to a too small AMR so that in the particular case of (Ga,Mn)As, extrinsic mechanism (i.e. anisotropy of *τ*) is dominant. The main source of scattering, magnetic atoms (manganese) substituting for cations of the host GaAs lattice, features magnetic and non-magnetic part (their ratio is described by parameter *α*_*sc*_) and while analytical estimates using equation (2.6) such as

$$\text{AMR}=-\frac{20\alpha_{sc}^{2}-1}{24\alpha_{sc}^{4}-2\alpha_{sc}^{2}+1},$$

can be obtained under simplifying assumptions, the full model shown in figure 5 reproduces the measured [73] AMR well. Also, various combinations of scattering and SO effects in two-dimensional electron gases have been explored: extrinsic anisotropy in Dirac fermions [74] or Rashba system [14,66,75].

Turning our attention back to transition metals (see tab. II in [1] for a list of material systems), two important publications should be mentioned. Mott [76] proposed that resistance in metals at high temperatures mainly depends on the scattering of 4s electrons into 3d states. At low temperature, the d-states are mainly populated, so that the main scattering is due to s-s-scattering and the resistivity is significantly lower. Smit applied this idea first to AMR [69] and proposed that the AMR can be only due to spin–orbit interaction (i.e. neglecting the possibility of intrinsic AMR), should always be positive and explained the larger AMR measured in dilute alloys by scattering due to foreign ferromagnetic atoms, where in simple transition metals (e.g. Ni) it is due to non-magnetic ions, lattice vibration or irregular stress. The foreign ferromagnetic atoms are supposed to have a larger effect on AMR than the other scattering effects, which also causes the AMR to decrease with increasing temperature (since lattice vibrations are becoming a more dominant contribution in resistance at higher temperatures) [69].

#### 2.2.2. Ab initio models

Most materials lack the simplicity of electronic structure which would render the construction of its effective model practicable. Band structure can nevertheless be obtained by ab initio methods (DFT or beyond) and should the AMR be dominated by intrinsic mechanism, plasma frequency for different magnetization directions can be calculated. Alternatively, conductivity can be obtained using Green’s functions *G* = *G*^+^(*E*_*F*_) in Kubo formula [77]

2.8 $$\sigma_{\mu \nu }(E)=\frac{e^{2}\hbar }{\pi V}\text{Tr }\langle v_{\mu }\text{Im }Gv_{\nu }\text{Im }G\rangle ,$$

by replacing the disorder average with $\tilde{G}v_{\nu }\tilde{G}v_{\mu }$ and ${\tilde{G}}^{-1}=E-H-i\Gamma$ with constant Γ (which in the limit Γ→0 drops out from the expression for AMR). When extrinsic mechanisms of AMR are important, a better treatment of scattering is needed and self-energy Σ (whereas Im Σ=Γ) must also be calculated by ab initio techniques.

The first attempt at such calculation has been undertaken by Banhart & Ebert [77] who employed the coherent potential approximation (CPA), but AMR as a function of *x* (fig. 1 in that work) was overestimated. Further refinements were made [78] and more recent calculations of Fe_*x*_Ni_1−*x*_ achieve a nearly quantitative agreement [79] to experimental AMR values. A different approach, based on modelling the system by layers also reproduces well [80] the experimental data on permalloy or Fe–Co [81] systems. Temperature-dependent AMR has now also been studied [82]. Beyond this material, cobalt alloys (with Pt or Pd [45]) and nickel alloyed with Cu or Cr [83] were studied, to give two examples among many. AMR in permalloy doped by selected transition metals (see fig. 2 in [82]) agrees reasonably well with ab initio calculations, with the exception of doping by gold, but it is presently unclear whether this is a failure of CPA (in this particular case) or an experimental issue [84]. Recently, it has been argued (based on the same theoretical technique) that in iron cobalt [13] the AMR is driven by intrinsic mechanism.

### 2.3. Further remarks

We conclude this section with several theoretical remarks before we proceed to the discussion of AMR in particular materials.

*Hexagonal systems.* In cubic systems, the resistivity tensor reduces to a number *ρ*_0_ (i.e. it is proportional to the identity matrix); we will now show that the same is true also for hexagonal systems. Assume that $\hat{x}$ is parallel to one of the sides of the hexagon. The two components of the resistivity tensor are denoted as $\rho_{∥}$ and *ρ*_⊥_ again. Then, if the tensor is rotated by an angle *θ*, its new form equals the form presented in equation (1.5). In a hexagonal system, a rotation of *θ* = *π*/3 is a symmetry operation and must not alter its properties. In this case, the zero off-diagonal elements must be conserved. In order to fulfil the equation 0 = (1/2)Δ*ρ*sin (2*π*/3), we have to demand $\Delta \rho =\rho_{∥}-\rho_{⊥}=0$. Plugging this into equation (1.5), the resulting resistivity tensor *ρ* = *ρ*_0_ · I_2_. This is not to say that AMR in hexagonal systems follows the same phenomenology as for example in tetragonal systems; however, it does mean that any observed anisotropy of in-plane transport (e.g. in NiAs-type antiferromagnet MnTe [62]) must be induced by magnetic order rather than by the hexagonal crystal structure.

**Figure 6.**
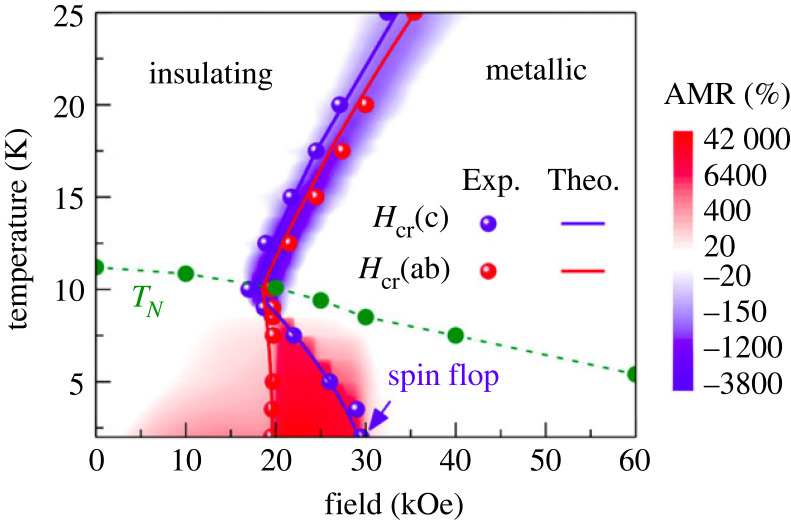
The magnitude of AMR in antiferromagnetic EuTe_2_ where the band structure changes from an insulator to semi-metal depending on the configuration of magnetic moments (which can be manipulated by an applied magnetic field). Reproduced from Yang *et al.* [87].

*Metal-to-insulator transition.* A very large change of electric conductivity can be achieved by tuning the system between metallic and insulating regimes: the typical system being vanadium dioxide [85]. While such typical MIT behaviour is unrelated to magnetism, proposals of magnetic order-dependent gap opening have appeared for orthorhombic CuMnAs [86], and experimentally, semimetallic antiferromagnet EuTe_2_ discussed in §3.3 is the first system where the transition between low- and high-resistance states was achieved [87] by rotating the magnetic moments as the phase diagram in figure 6 shows. This effect can be understood as the extreme case of intrinsic AMR: rather than deforming the Fermi surface (FS) slightly by rotating the magnetic moments, the FS disappears altogether. A related effect can also occur in magnetic topological insulators, see §4.2.4.

*Relative and absolute AMR.* It is customary to evaluate the AMR in relative terms. This makes good sense for extrinsic AMR where both *ρ*_0_ and Δ*ρ*_0_ are proportional to the density of scatterers *n*_*imp*_ and the ratio (equation (1.1)) is then independent of *n*_*imp*_. Fig. 4 in [4] demonstrates that this may be true for a large group of samples. On the other hand, when resistivity comprises two additive parts (in the spirit of Matthiessen’s rule) where one is anisotropic and the other is not, it is more meaningful to focus on absolute difference of resistivities for magnetic moments parallel and perpendicular to current. This is also the case for polycrystalline samples where the isotropic part of resistivity is due to scattering on grain boundaries: a suitable approach is then the Fuchs–Sondheimer theory discussed e.g. in the introduction of Rijks *et al.* [88].

## 3. Materials

### 3.1. Elemental transition metals and their alloys

The first observation of AMR was made in iron and nickel with cobalt following (see §1.2 for the history), and understandably, the first microscopic theories, therefore, aimed at elemental FMs. The first step beyond the quantification of the AMR ratio on the level of equation (1.1) was to analyse individual symmetry contributions to the AMR [5] (as given by equation (2.4) or equation (2.2) nowadays), and next, their temperature dependence was determined [23,89]. Other papers on these materials are discussed in §2.2.2 since the results of them are outdated by now, but of historical importance in the development of models. In the following, the most interesting results are discussed and typical AMR values are listed in table 1. There is some scatter in the values of AMR, whose origin cannot be conclusively identified, since part of the information is lacking in some of the studies. So it is unclear whether in all studies the saturation magnetization is reached, what are the crystal structure and crystalline quality, and in very thin films, surface scattering can even play a role. Important observables to watch are the sign of the AMR and the order of magnitude of the values. As discussed previously in §2.3, in the case that only relative AMR values are stated, it is unclear whether certain AMR values are due to scattering or the background resistivity.

State-of-the-art reports of AMR in the three transition metals are for iron thin layers by van Gorkom *et al.* [23] and for nickel films by Xiao *et al.* [90]. While these two metals are cubic (bcc iron belongs to space group Im3¯m, fcc nickel to $\mathrm{Fm}\bar{3}m$), the situation is somewhat more complex for cobalt which exists in the hcp [91] (space group P6_3_/mmc) and fcc [92] phases. In polycrystalline samples [24], the AMR is a factor of about 1.8 larger for fcc than for hcp (hexagonal close packing). This behaviour was explained by differences in (calculated) DOS at the Fermi level. The hcp-Co AMR is reported to lie between 1.14% and 1.23% and for predominantly fcc-Co samples the span is 1.73–2.19%.

Polycrystalline Co has a dominant intrinsic AMR contribution, which was shown by frequency-dependent studies [15] on ac-AMR (see §4.2 for details on the ac-AMR method). In the same study, it was also shown experimentally that polycrystalline Ni and the alloys Ni_*x*_Fe_(100−*x*)_ with *x* = 50 and *x* = 81 (permalloy) have a negligible intrinsic contribution and plasma frequency calculations indicate a similar behaviour in single-crystalline materials. For the single-crystalline case, experimental confirmation is still required. For iron, such investigations are lacking entirely and it is thus unknown whether the AMR in Fe is caused by extrinsic or intrinsic contributions.

In the analysis of Döring [5] based on equation (2.4) or equation (2.2), fourfold signals were also identified in single-crystalline nickel. However, the reporting of higher-order signals in these basic TMs is rare [90] and usually only twofold signals are reported. In some studies, deviations from twofold AMR are accounted for by MCA using Stoner–Wohlfarth approaches as for example was reported by Miao *et al.* [93] in single-crystalline Co and polycrystalline Fe_20_Ni_80_ as well as in epitaxial Fe_30_Co_70_ thin films [33].

Alloys offer a vast field for research on AMR since the effect can be increased significantly, as compared with the pure TMs, by tuning their composition. Our discussion of alloys is split into two categories: first, the three basic TMs with small amounts of TM impurities are discussed and second, we focus on alloys made from a combination of the three basic TMs. The best-known example of the second category is permalloy (Ni_80_Fe_20_). Typical values of AMR ratios are listed for the alloys in table 1 as well.

A comprehensive work on the first category of alloys, nickel with TM impurities, is Jaoul *et al.* [16]. An important characteristic of these impurities is the virtual bound state (VBS); when the VBS appears [94] (for example with V, Cr, Os or Ir) both positive and negative AMR was measured, and otherwise, the AMR remains positive (this was the case with Mn, Fe, Co, Pd, Cu, Zn, Al, Si, Sn and Au where the VBS does not appear). This was attributed to the effect of the *L*_*z*_ *S*_*z*_ operator of the spin–orbit interaction on the VBS, which was included in the description of AMR by adding the term +3*βα*/(*α* + 1) to equation (2.7), where *β* encrypts the effect of the *L*_*z*_ *S*_*z*_ term. It can be positive or negative, thus the AMR can show both signs. Please note, that this explanation for negative AMR is consistent with the more recent and elaborate one given by Kokado and Tsunoda [27] (see §1.2). Contrary to the latter ones, the extension of equation (2.7) by Jaoul is limited to strong FMs and is not capable of describing e.g. features of half-metals such as spin-dependent effective mass. In another study by McGuire *et al*. [17], robust negative AMR up to RT was achieved by considering Ir as an impurity in various hosts such as nickel, cobalt, iron and in certain alloys of these three.

In the second category of alloys, we find the combinations FeCo, CoNi and NiFe as well as FeCoNi in diverse compositions. AMR in these alloys is robust and typically one order of magnitude larger than in the pure TM, as can be seen in table 1. Many publications focus on AMR measurements for different compositions and track the dependency of AMR on the concentration of a certain element. Of special interest is permalloy, which shows not only a large AMR but is also used in a number of industrial applications, for example in magnetic readout heads. The interest of industry is due to its nearly zero magnetostriction and high magnetic permeability.

Composition-dependent studies of the AMR ratio in the nickel-rich alloys Fe_*x*_Ni_1−*x*_, Co_*x*_Ni_1−*x*_ and (Co_*x*_Ni_1−*x*_)_86_Fe_14_ were carried out by Ishio *et al.* [19] and in the iron-rich alloys NiFe and FeCo by Berger *et al.* [20]. In the first case (bulk monocrystals of Ni-rich alloys), Ishio *et al.* report the AMR ratio for two different current directions [001] (which they call *K*_1_) and [111] (which they call *K*_2_), and their large difference implies that, in terms of equation (2.4), the crystalline AMR is larger than the more commonly measured non-crystalline term in equation (1.1). With the caveat of the residual resistivity being very low, we note that extremal values of (relative) AMR, achieved for (Ni_80_Co_20_)_86_Fe_14_ reach $K_{1}=+68\%$ and $K_{2}=-32\%$. For *K*_2_ there is an increase leading to a sign change to positive values with increasing Fe and Co [19]. This is consistent with other measurements reporting AMR of up to 50% in NiCoFe alloys with a maximum at Ni_80_Co_20_Fe_5_ [95]. In the FeNi alloys, a maximum AMR (*K*_1_) of approximately 35% is achieved at approximately 10–15% Fe. Permalloy shows an AMR of 25% [19]. In the second case of the iron-based alloys by Berger *et al.* [20], the AMR is split into an impurity-based AMR contribution (Δ*ρ*/*ρ*)_*im*_ and a phonon-based contribution (Δ*ρ*/*ρ*)_*ph*_. Both contributions are indiviually plotted vs. the iron concentration. (Δ*ρ*/*ρ*)_*ph*_ is positive for the case of weak electron scattering in Fe–Co and negative in the case of strong, resonant electron scattering in the other alloys. The impurity contribution is always positive and larger for the strong scattering. A maximal AMR of approximately 16% is found for permalloy. A more recent study [93] reports only few per cent AMR for sputtered Ni_80_Fe_20_ films, but in absolute terms, i.e. $R_{∥}-R_{⊥}$ in equation (1.1), the anisotropy is similar in both samples; here, the buffer layer thickness also plays role [96] most likely through changing the background resistivity, as discussed in §2.3. AMR in epitaxial Fe_30_Co_70_ was shown to have strong crystalline terms [33]. More alloys involving transition metals are discussed in §3.6.

**Figure 7.**
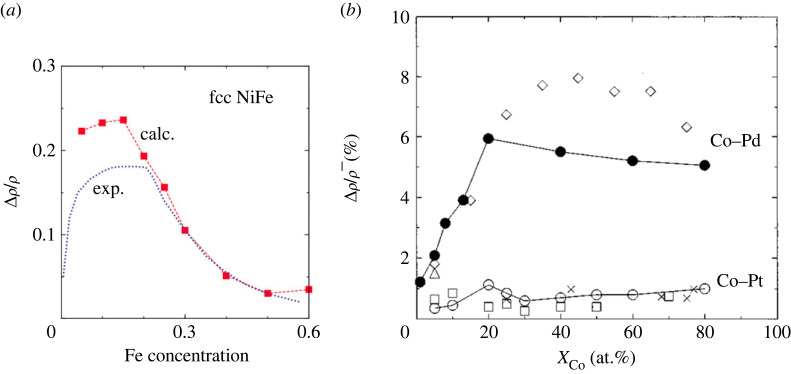
AMR in alloys (*a*) nickel–iron, (*b*) cobalt with non-magnetic elements. Reproduced from (*a*) Turek *et al.* [79] and (*b*) Ebert *et al.* [45].

A comparison of experimental data and CPA calculations is given in figure 7*a*, where the AMR ratio is calculated for fcc NiFe alloys dependent on the Fe concentration. Especially for concentrations larger than 0.15, the calculations describe the experimental data almost perfectly. Calculations of dilute NiFe thin film, wires and FM/non-magnetic/FM multilayers using Boltzmann equation with RTA and a two-current model are carried out by Rijks *et al.* [88].

### 3.2. Dilute magnetic semiconductors

A completely different perspective of AMR is offered by the DMSs: magnetism and transport properties can be tuned in these systems to some extent independently. Our understanding of the electronic structure in DMSs relies on the solid knowledge about III–V (and other) systems such as GaAs combined with the substitutional effect of a magnetic element (typically manganese), whereas coupling between localized magnetic moments (provided in that case by 3*d*^5^ electrons) is mediated by delocalized carriers [72]. The key parameter is the acceptor (in the case of III–V:Mn) binding energy *E*_0_ and also its physical origin [97] indirectly influences the magnetotransport mechanism.

Given the appreciable spin–orbit interaction in GaAs (Δ_SO_ = 0.34 eV) and basically metallic conduction (fig. 32 in [72]), AMR could have been anticipated to occur in (Ga,Mn)As. Indeed, the first report of AMR in (Ga,Mn)As [98] has soon been followed by more detailed studies [73,99] and new ideas keep appearing (co-doping by lithium [100] or As/Sb substitution [101,102]). These studies allowed to explore the AMR under continuous variation of band structure parameters and filling as well as of strain [10,54].

**Figure 8.**
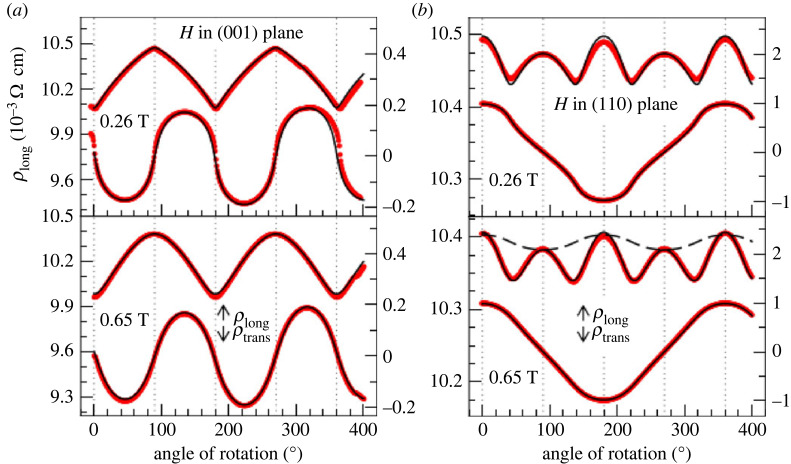
The data (red thick lines) and fit (black thin line) of the longitudinal resistivity *ρ*_long_ (upper line in every plot) for the current direction along [110]. The magnetic field of 0.26 and 0.65 T is rotated in the (001) and (110) plane, respectively. The dashed line in the lower panel of (*b*) refers to an attempt of fitting the data to cos (2*ϕ*), which is clearly insufficient. The lower lines in every plot are the transversal resistivity. Reproduced detail from fig. 7 in [54].

Research on AMR in DMSs has pushed the understanding from ‘complicated to simple’ concepts: idealized *sd*-models [1] gave way to a semiquantitative description [103] where the intrinsic and extrinsic sources of AMR [15] could be separated (see §1.4 and then the detailed discussion of microscopic models in §2). It should be noted that also intrinsic AHE could be explored in detail [65] in this class of materials. These models were quite successful in describing the dominating non-crystalline AMR but fourfold crystalline AMR, whose contribution can be clearly seen in figure 8 (an SW1 model was used to analyse magnetotransport in (Ga,Mn)As in [54]) remained beyond reach [28]. In the lower panel of figure 8*b* it can be clearly seen that an attempt of fitting the angular dependence to terms without fourfold terms leads to insufficient agreement. Currently, interest in the once very popular (Ga,Mn)As subsides, since the prospects for the RT magnetism [104] remain unfulfilled. Nevertheless, Mn-doped III–V semiconductors remain a good test-bed for exploring transport phenomena in materials with tunable magnetic properties.

Despite the versatility of this material class, not much attention was given to other DMS: twofold and eightfold AMR were reported in a 10 nm film of (In,Fe)As in [63]. In a 100 nm film of the same material, the eightfold component was missing, which was attributed to higher electron concentration. Yet this claim is not supported by microscopic calculations and, together with the very small magnitude of AMR and low electron concentration, this may well be a hint that it is not the absence of *sd*-scattering at the Fermi surface [63] but issues with sample quality that lead to this unusual behaviour. Better established materials, in terms of sample quality, such as (Cd,Mn)Te, still suffer from too low carrier concentration [64], and even if the regime of metallic conduction is reached [105], only MR (rather than its anisotropy) is measured, and transport mechanisms seem to be less well-established than in the case of (Ga,Mn)As. These systems also occasionally suffer from the formation of multiple phases [106]. Finally, we would like to mention magnetically doped *A*_2_*B*_3_ systems (where *A* is either Bi or Sb and *B* is Se or Te) [107,108] as well as magnetically doped ZnO [109], whereas in the latter, the mechanism of magnetic state formation is complicated and can even be achieved by hydrogenation of ZnO [110].

### 3.3. Antiferromagnets

While ferromagnetism has been a known phenomenon since ancient times, its counterpart antiferromagnetism was introduced no earlier than 1933 by Landau [111]. It is little surprising then, that AMR in this material class has only recently been investigated. About 10 years ago, the first studies appeared reporting AMR in antiferromagnetic (AFM) Sr_2_IrO_4_ [112,113] (space group I4_1_/acd) and in FeRh (space group $\mathrm{Pm}\bar{3}m$) which undergoes a transition from AFM to FM [114,115]. In recent years, the class of AFM materials has received more attention due to the development of AFM spintronics [116]. The hope is to revolutionize spintronic applications by making use of the advantageous properties of AFMs such as robustness against magnetic field perturbations, the lack of a stray field or ultrafast dynamics. A prototypical magnetic memory was developed using CuMnAs (see §4.3), and the transversal component of AMR (also called the planar Hall effect) was used as readout [117]. As tetragonal CuMnAs (space group P4/nmm) has thus become a popular AFM material [118], its properties came under intense scrutiny; microscopic mechanism of its AMR is, however, far from clear [53]: multiple kinds of impurities lead to AMR, which is comparable to experiment. On the other hand, intrinsic AMR linked to gap opening controlled by Néel vector orientation was proposed [86] to occur for the orthorhombic phase [119] of CuMnAs, which is similar [120] to another AFM metal: Mn_2_Au.

**Figure 9.**
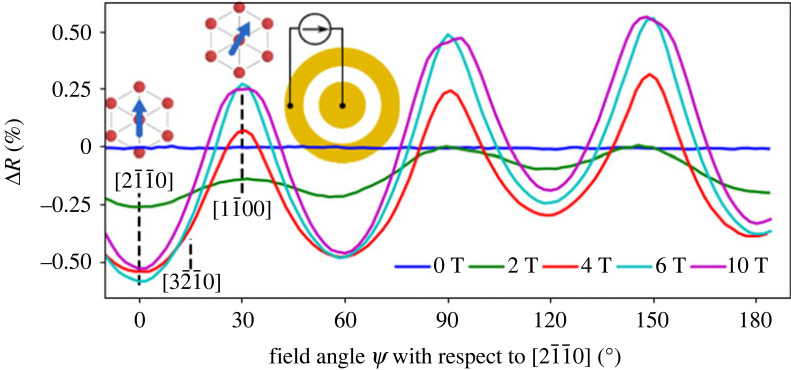
Crystalline AMR was measured in MnTe for different field strengths using a Corbino geometry. The AMR shows a sixfold symmetry, which can be expected for the crystalline AMR in a hexagonal material. Reproduced from Kriegner *et al.* [62].

Another material which is a candidate for magnetic memory is MnTe: next to a robust, continuously varying AMR signal suitable as readout for AFM states, stability of the AFM states against perturbing magnetic field itself was shown using zf-AMR [51]: resistivity is measured in zero magnetic field at low temperatures after the sample is field-cooled in a *writing field*. After taking a data point, the sample is heated up again and the procedure is repeated for another orientation of the writing field. Repeating this for a continuous rotation of writing fields yields a periodically zf-AMR signal resembling the conventional AMR. Furthermore, in the experiment it was shown that for a writing field of 2 T, the zf-AMR is multi-stable against perturbations from magnetic fields of 1 T or less. Hence, the possibility of writing and readout combined with robustness against perturbing fields makes it an excellent candidate for a spintronic device [51]. Also, crystalline AMR measured in the Corbino geometry shown in figure 9 shows a strong cos (6φ) component due to the hexagonal crystalline structure of MnTe [62].

In the AFM Mott-insulator Sr_2_IrO_4_, point-contact measurements in a single-crystalline bulk sample at liquid nitrogen temperature yielded a field-dependent transition from fourfold AMR (low field) to twofold AMR (high field). The fourfold AMR was interpreted as crystalline AMR reflecting the tetragonal crystal structure of the single-crystalline sample, while the transition to twofold AMR was due to canting of AFM moments. The AMR ratio shows a maximum of 14% at a field of 120 mT. The large AMR has been attributed to large SOC in this 5d oxide [113]. In another experiment, AMR in a Sr_2_IrO_4_ film is studied by using a SIO/La_2/3_Sr_1/3_MnO_3_ (LSMO) heterostructure. The ferromagnetic LSMO is used to control the reorientation of AFM spin-axis via exchange spring effect. The AMR at low temperatures (*T* = 4.2 K) is showing a fourfold behaviour, while at intermediate temperatures (*T* = 40 K) no AMR signal was detected, and at higher temperatures (*T* = 200 K) the AMR is dominated by the twofold AMR of the FM LSMO [112].

An AFM memory in FeRh was proposed by Marti *et al.* [114] where field-cooling is used to write a magnetic state and AMR is used as readout. Similarly to MnTe [51], the memory shows a certain insensitivity against perturbing fields [114]. RhFe undergoes a FM–AFM transition. It is antiferromagnetic below *T*_*N*_ = 370 K and ferromagnetic between *T*_*N*_ and *T*_*C*_ = 670 K. Transport was investigated for both phases using first-principle calculations (relativistic TB-LMTO). AMR exists in both the FM and the AFM phase and was stated to be in a range of up to 2% depending on the Rh-content. The AMR in the AFM phase is larger for most of the investigated compositions [115].

A rather special case is single crystals of AFM EuTe_2_ where a peak value of 40 000% at 2 K and 22 kOe (2.2 T) is achieved due to a metal–insulator phase transition (MIT). Since the MIT shows different critical fields for the *ab*-plane and the *c*-axis, the AMR becomes colossal for applied field values between the in-plane and the out-of-plane critical fields leading to a several order of magnitude change in resistivity for rotating the magnetic field. Band structure calculations confirmed this behaviour. AMR for fields and temperatures entirely within one phase of EuTe_2_ (metallic or insulating) is in an order of magnitude of less than 20% and thus comparable to other materials [87].

Furthermore, we remark that (collinear) ferrimagnets can be considered to belong to this group too [121], since similarly to the AFM, they can have two MSLs, with the difference that the magnetic moment is not fully compensated. Finally, AMR in non-collinear AFMs is a rather novel topic and will be discussed in §4.2.3.

### 3.4. Heusler alloys

*Introduction.* Heusler compounds exhibit a large variety of remarkable properties, as for example ferromagnetism and antiferromagnetism, thermoelectricity, high spin-polarization, superconductivity and topological features [122]. In general, they are cubic (space group $\mathrm{Fm}\bar{3}m$) and their formula is X_2_YZ, where X and Y are transition metals and Z is a main group element. X is more electropositive than Y. If X and Y are exchanged, the material is called an inverse-Heusler. There are so-called half-Heuslers, which are given by the formula XYZ [122]. In general, Heusler compounds have a cubic crystal structure, which can occur in different variations. The first Heusler compound was Cu_2_MnSn, discovered already in 1903, which was a surprise because it was ferromagnetic while its components are not [123].

Despite the generality of its definition, a large body of research is focused on cobalt-based Heusler alloys (thus Co_2_YZ and Y is typically Mn, Fe or a lighter 3d element), since they generally show important features interesting for potential spintronics applications, such as relatively high Curie temperatures, half-metallicity, large magnetotransport effects and many more.

*Co-based Heusler compounds.* There is some degree of scatter in the AMR values reported for Co-based Heusler alloys. For example the values for Co_2_MnGa were found to lie between −2.5% and +0.75% depending on the current direction and precise stoichiometry [32] as these dependences can be analysed in terms of crystalline and non-crystalline terms as in figure 4 (see discussion later in this section). However, a meaningful comparison between the epitaxial [32] and sputtered [12] samples requires also the knowledge of background resistivity [31] proportional to *R*_0_ from equation (1.1).

**Figure 10.**
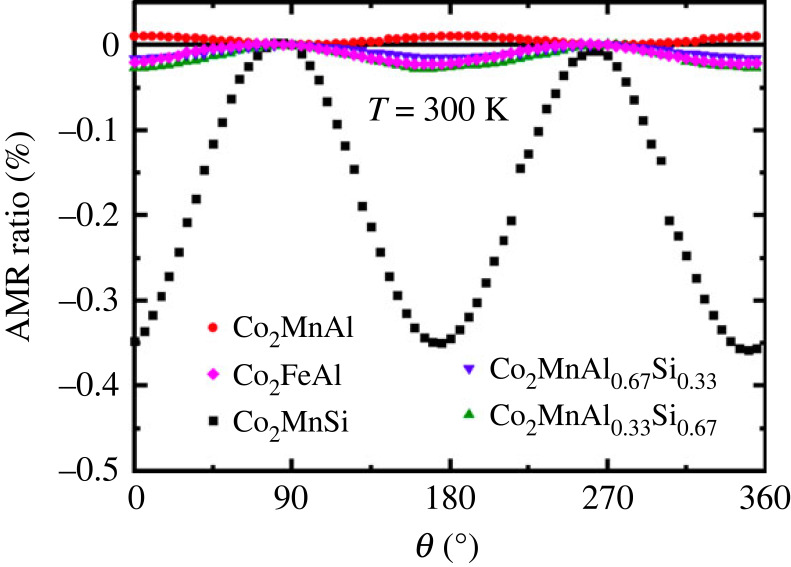
AMR in Co_2_MnAl (CMA), Co_2_FeAl, Co_2_MnSi (CMS) and Co_2_MnAl_*x*_Si_1−*x*_ for x=0.33 and 0.67. The AMR for CMS has the largest magnitude. All materials exhibit negative AMR apart from CMA. The order of magnitude of the AMR is in agreement with other studies of Co-based Heusler alloys. Reproduced from Breidenbach *et al.* [124].

On the other hand, once the current direction is fixed (here, along [110] crystallographic direction) we often arrive at similar characteristics of AMR even for different compounds: measurements of Co_2_MnGa by Ritzinger *et al.* [12] and of Co_2_FeAl by Althammer [9] show negative AMR which decreases with temperature and is quite small (approx. 0.1–0.2%). Several other examples are given in figure 10.

**Figure 11.**
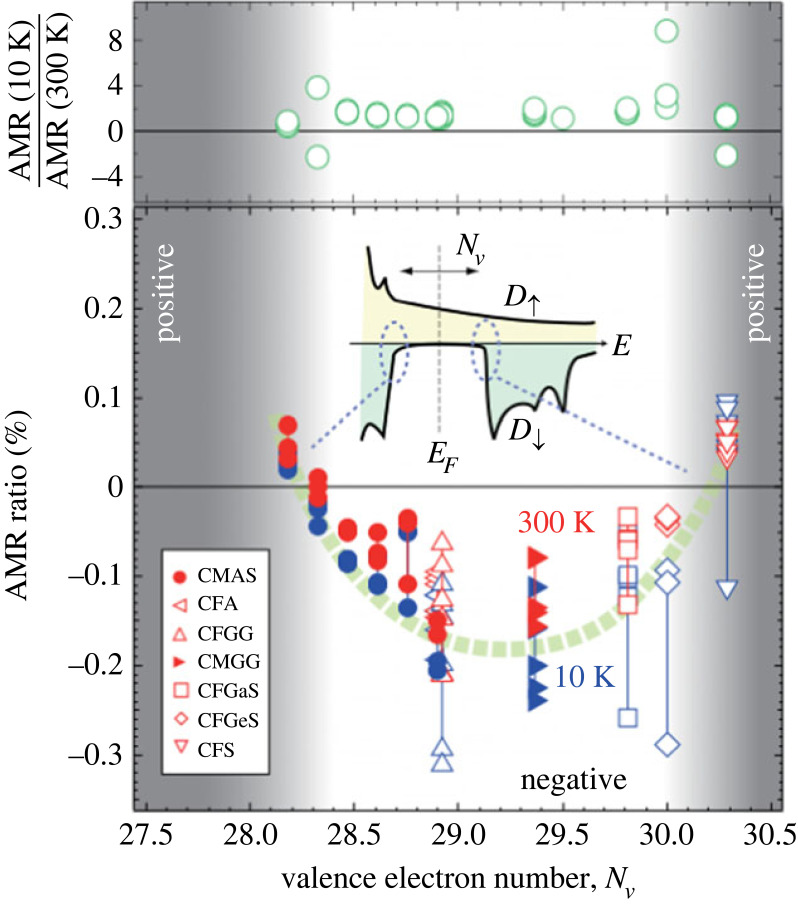
Valence electron number *N*_*V*_ dependence of AMR ratio in all Co_2_MnZ and Co_2_FeZ films. The inset shows the respective density of states. The upper part shows the ratio of AMR ratios at 10–300 K. Reproduced from Sakuraba *et al.* [125].

Investigation of AMR in Co_2_FeZ and Co_2_MnZ with Z = (Al, Si, Ge, Ga) and current along [110] found both negative and positive AMR, depending on the total number of valence electrons *N*_*v*_. If that number was between 28.2 and 30.3, a negative AMR was reported, otherwise a positive. According to band structure calculations, in between *N*_*V*_ of 28.2 and 30.3, it corresponds to half-metallicity [125] as can be seen in figure 11. The reported AMR ratios in this paper are relatively small in comparison with other papers. An equivalent result was achieved in Co_2_Fe_*x*_Mn_1−*x*_Si: Here the AMR is negative for *x* ≤ 0.6 and positive for *x* ≥ 0.8, which is explained by a transition from minority conduction to majority conduction and thus interpreted as a possible sign for half-metallicity as well [30]. Similarly in Co_2_FeSi, the AMR ratio was determined for different samples distinguished by their annealing temperature: above $600^{\circ }C$ the AMR is negative, up to $600^{\circ }C$ it is positive with the same explanation as before [35]. The AMR ratio in Co_2_(Fe − Mn)Si, Co_2_(Fe − Mn)(Al − Si) and Co_2_(Fe − Mn)Al was reported to be approximately −0.2% for low and RT [126].

**Figure 12.**
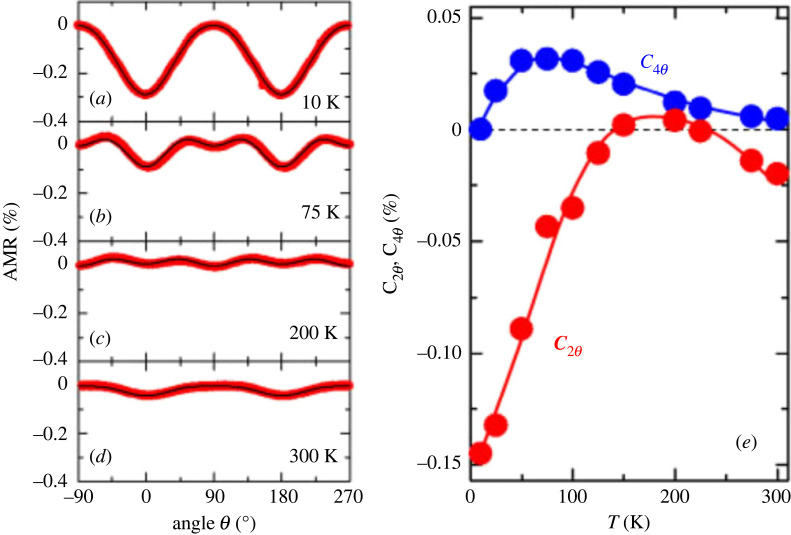
AMR in Co_2_MnSi. (*a*–*d*) AMR for different temperatures between 10 and 300 K, (*e*) temperature evolution of the two- and the fourfold Fourier-component of the AMR. Reproduced from Oogane *et al.* [127].

While the majority of studies focus on AMR ratio and sign, the symmetry of these compounds is also a puzzling topic: in Co_2_MnGa [12], Co_2_FeAl [9] and Co_2_MnSi [127], the AMR showed a complex signal comprising non-crystalline and crystalline terms. However, the division into non-crystalline and crystalline is usually not made and the AMR is only described in terms of cos (4*ϕ*) and cos (2*ϕ*) contributions (*ϕ* being some angle of rotation). The higher-order symmetries make AMR in these materials much more complex as e.g. in simple transition metals, where normally only twofold symmetries are found. An example of such a rather complex signal in Heusler compounds can be found in figure 12.

The fourfold contributions in these signals are too strong to be ascribed to MCA solely. In a theoretical study by Kokado & Tsunoda [61], it was suggested that a tetragonal distortion of the crystal structure can introduce such a fourfold crystalline AMR contribution. Please note, that the Heusler alloys *per se* have a cubic crystal structure, but in thin films the substrate usually introduces a small tetragonal distortion. Still, despite this explanation being plausible, the complex temperature dependence of the twofold and fourfold contributions [9,12] asks for further investigation.

In conclusion, a couple of observations can be made for AMR in Co-based Heusler alloys: firstly, the AMR ratio is generally decreasing with increasing temperature, as expected. Secondly, the AMR is very small, often well below 1% and only in some specific configurations (low temperature, favourable stoichiometry) it reaches up to approximately 2%. Thirdly, the AMR ratio given by equation (1.1) is usually negative; however, it can be tuned to be positive. This behaviour can be seen consistently in various studies and appears to be a general property of this material class of the Co-based Heusler alloys. Various ‘phenomenological’ explanations for the sign change of AMR are given, e.g. dependence on annealing temperature, Fe-content, Co-content, current direction and *N*_*v*_. These explanations are rather diverging and not allowing for a consistent conclusion. On a microscopic level, however, the various studies can be summarized quite well: as long as the compounds are having a half-metallic character/showing minority conduction, the AMR is negative. In the case of majority conduction and metals not fully polarized on the Fermi level, the AMR becomes positive. It appears to be the case that the Co-based Heuslers investigated here are all *by default* (= in an ideal configuration) half-metallic, but can be all tuned to lose this half-metallic character (this tuning was done by considering the phenomenological aspects, such as annealing temperature). The theoretical model used to explain it was developed by Kokado & Tsunoda [27]. It is not yet clear whether the given data has universal character.

*Non-Co-based Heusler and semi-Heusler compounds.* Just as with transition metal alloys, Heusler materials [128] span a vast range of compounds: magnetic Heusler alloys include NiMnSb [129] or Ru_2_Mn_1−*x*_Fe_*x*_Ge. The latter is a ferromagnet for *x* = 1 (no Mn) and an antiferromagnet for *x* = 0 (no Fe). For *x* = 0.5 an anisotropy in the MR is observed with a MR of −4% and +2% under parallel and perpendicular configurations of the applied field and applied current, respectively. It was speculated that this (anisotropic) MR might stem from a random alignment of ferromagnetic domains. For *x* = 0 and *x* = 1 no MR was found [49].

### 3.5. Two-dimensional electron gases

*Introduction.* A two-dimensional electron gas (2DEG) can form on various interfaces: the surface of liquid helium, classical semiconductor heterostructures or certain transition metal oxide interfaces (TMOI). The textbook example of such a TMOI is a SrTiO_3_/LaAlO_3_ (STO/LAO) interface, where the two perovskites individually are non-magnetic insulators [130]. The research interest in TMOIs can be broadly speaking divided into three categories: (i) general understanding of the electronic structure, magnetism and related effects, (ii) understanding of the superconductivity [131–133] (transition temperature is typically of the order of 0.1 K [134]), and (iii) possible development of applications, such as quantum-matter heterostructures [135]. Regarding AMR in TMOI-hosted 2DEGs [136], it is important to distinguish if the transport anisotropy occurs due to orbital effects [137] as discussed in §1.4 on general level, or if it is indeed related to magnetism. Hysteretic magnetization loops observed in STO/LAO structures grown at suitable oxygen pressure [138] can be taken as a hint of the latter, yet the MRs shown in fig. 3 of [138] clearly show that even here, the orbital effects are strong. On the other hand, longitudinal and transversal MR showing similar behaviour of LTO/STO (LTO = LaTiO_3_) at stronger magnetic fields can be taken as an argument that the latter are *not* dominant (compare fig. 2*d*,*e* in [134]). The focus of many publications lies on LAO/STO interfaces, whose results are discussed in the following. A summary of AMR in other TMOI-hosted 2DEGs (including LTO/STO [134]) can be found at the end of this section.

**Figure 13.**
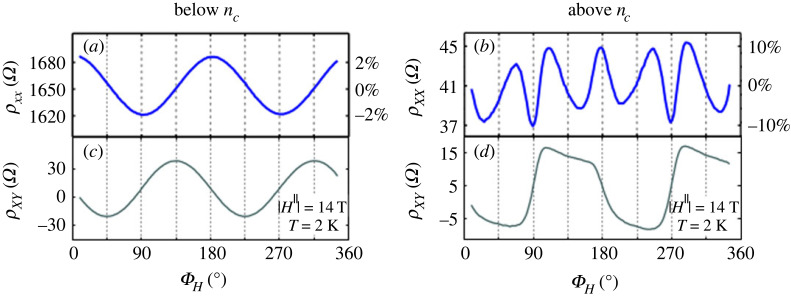
(*a*,*c*) AMR in a 2DEG on LAO/STO interface for electron density below (*a*) and above (*c*) the critical value. Reproduced from Joshua *et al.* [139].

*AMR in LAO/STO.* On a qualitative level, the AMR of a 2DEG at the LAO/STO interface can exhibit two types of behaviour, see figure 13. This was attributed to a phase transition when going to low temperatures *T* and high carrier densities *n*. A positive and twofold AMR was found for temperatures *T* > 35 K, while for lower *T* and higher *n*, a negative AMR and higher orders up to sixfold symmetry in (111) and (110) interfaces [131,139–141] were found.

The absolute value of the AMR ratio is not the major point of discussion and should be taken with maximum caution, due to its strong dependence on temperature [142], current density (AMR increasing with increasing *n* [140]), *B*-field strength [141,142] and many open points in the understanding of the inner workings of AMR in these materials. The AMR was reported to be larger in the low-*T* high-*n* phase (approx. 2% below and approx. 10% above the critical *n*) [139]. A large value of 110% was reported for some $\left[1\bar{1}0\right]$ oriented samples grown under low oxygen pressure with *B* = 9 T [142], which was understood in terms of oxygen vacancies leading to stronger orbital polarizations and producing a more anisotropic FS which in turn leads to larger AMR [142]. Also, the band structure and thus the FS and the AMR are strongly dependent on the sample orientation [142] and oxygen pressure during growth.

**Figure 14.**
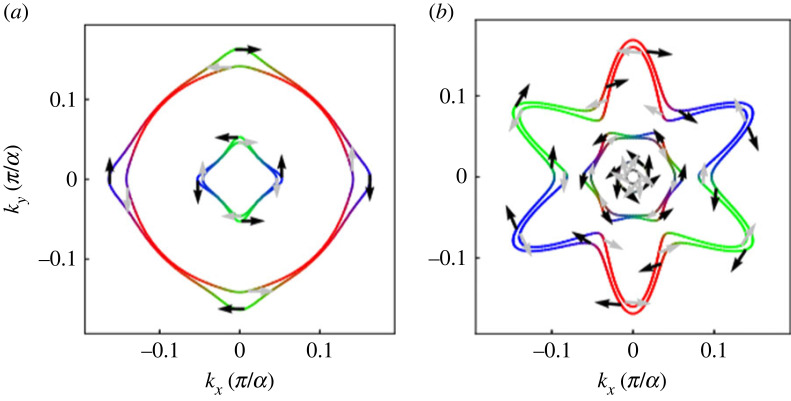
Fermi surfaces for a six-band model spin–orbit coupled 2DEGs at zero magnetic field, with colours indicating orbital content (*yz*-blue, *zx*-green, *xy*-red), and Rashba spin texture indicated by black/grey arrows for opposite chiralities. (*a*) (001) 2DEG and (*b*) (111) 2DEG. Both Fermi surfaces are highly anisotropic. Reproduced from [143].

In calculations, the AMR is frequently linked to a strong anisotropy of the FS as exemplified in figure 14 [137,139,142,143]. Although this means that the AMR is intrinsic, the distinction between intrinsic and extrinsic AMR in these studies is usually not made. The harmonics of the AMR, i.e. the strength of the twofold, fourfold and sixfold are not directly linked to the symmetry of the FS [143].

The electronic structure at the FS is different between the low-*n* and high-*n* regimes [137,143] and is also sensitive to the crystallographic direction of interface [141,143]. The anisotropy appears to be driven by interband scattering, which is suppressed in the low-*n* regime [143]. The *t*_2*g*_-orbitals and broken inversion symmetry are generally a central part in the modelling of LAO/STO interfaces [137,139,141–143].

*Other materials.* Apart from the much-investigated LAO/STO interface, 2DEGs at a TMOI can be found in other material combinations, for example LVO/KTO (LaVO_3_/KTaO_3_) [144], LVO/STO [145], LTO/STO [134] and CZO/STO [146] interfaces where CZO stands for CaZrO_3_ (the last-mentioned system stands out by being non-polar without strain). AMR was only studied in the first two examples; sometimes, anisotropic data is shown [134] (longitudinal and transversal MR seperately, as mentioned before), although not being referred to as AMR in the respective publication. The results are shortly summarized in the following. Please note that the similarity of the 2DEGs in LTO/STO and CZO/STO do still suggest the existence of similar AMR phenomena, which have yet to be investigated.

In low-temperature measurements in (001)-interfaces of LVO/KTO [144] and LVO/STO [145] a low-field twofold AMR turned into a high-field fourfold AMR. In the case of a (111)-interface of LVO/STO the high-field AMR was sixfold. AMR in LVO/STO showed a strong field and temperature dependence. The larger fourfold AMR persisted up to 150 K while the sixfold AMR persisted up to 20 K, similar to the situation in LAO/STO [145]. While no profound explanation was given for the LVO/KTO interface [144], it was suggested that AMR in LVO/KTO is due to an anisotropic FS, similar to the situation in LAO/STO [145].

### 3.6. . . . and all the rest

The previous five sections of this section were devoted to the material classes showing the most important and remarkable results in the field of AMR. This is not nearly a complete picture of the universe of AMR. In the following section, we are going to discuss briefly the results of several other material classes.

*Fe-based alloys*. Apart from iron–cobalt and iron–nickel alloys which were discussed already in §3.1, Berger *et al*. [20] investigated AMR also in Fe–Cr and Fe–V and split the AMR contributions into parts due to phonon and impurity scattering (see also discussion of Berger *et al.* [20] below in the context of Co–Pd alloy). It was suggested that in alloys with strong scattering the AMR changes sign when the impurity scattering is maximal. According to this study, a change of 3d-DOS does not account for all of the observed behaviour.

In Fe_0.8_Ga_0.2_ it was found that the AMR is twofold and for in-plane (out-of-plane) configuration at a magnetic field of 500 mT (8 T) showed negative (positive) AMR. Interestingly, with increasing temperature the AMR is constant (decreasing). The AMR ratio is slightly larger than 0.1% (between *ca* 0.2% and 0.5%). The perfect twofold-shaped AMR curves were interpreted as a sign that saturation magnetization was reached [147].

Properties of NiFeCr alloys such as AMR ratio, low-temperature resistivity and *T*_*C*_ depending on the Cr concentration are listed in tab. 1 of [21]. A maximum AMR of 0.76% is found in the sample with the lowest Cr concentration of 2%. Increasing the chromium content leads to a rapid decrease of AMR ratios until the AMR almost vanishes for concentrations higher than 18%. Please note that this study is using the term *ferromagnetic anisotropy of resistivity* instead of AMR. The rapid decrease of the AMR ratio is accompanied by a drop of *T*_*C*_, from 778 K for 2% concentration to 48 K at 21% concentration [21].

*Other alloys or structures involving transition metals.* The AMR of Co–Pd alloys was investigated in [18] for various cobalt concentrations *x* and its temperature dependence was analysed in terms of Parker plots (as discussed in the introduction of Berger *et al.* [20]). A maximum ratio of almost 8% was reported at low temperatures for almost equal concentrations of Co and Pd. The results were interpreted with the framework of s-s- and s-d-scattering, splitting the resistivity into contributions of spin up and down, s-s and s-d-scattering, and phonon and impurity contribution to the AMR [18]. It can be seen as an extension of the theory of Campbell, Fert and Jaoul discussed in §3.1.

Calculated values of the AMR ratio and the residual resistivity of Co–Pd and Co–Pt alloys as a function of the cobalt-concentration are shown in figs. 5 and 3 of Ebert *et al.* [45], respectively. The values are compared with experimental values from various studies, which showed the accuracy of the calculation. In the case of Co–Pt the AMR reaches values of up to 1%, while in the Co–Pd case the AMR shows a maximum of 6% (calculation) or 8% (experiment). The AMR is starkly decreasing for very low Co-content [45]. Note that even for concentrations as low as 3% of cobalt, palladium alloys remain ferromagnetic [46] and the AMR can be reasonably modelled assuming |*J*| = 43 meV for the coupling between magnetic moments and highly conductive *s*-electrons.

The in-plane and out-of-plane AMR of nickel sandwiched by platinum was experimentally investigated and the symmetry of the AMR discussed [148]. The nickel films are fcc textures with a (111) surface and have a thickness between 2 and 50 nm, while the platinum layers are 5 and 3 nm thick. The in-plane AMR shows only twofold symmetry as expected for an isotropic polycrystalline sample. The out-of-plane AMR shows pronounced fourfold and sixfold symmetries for nickel thickness greater than or equal to 6 nm. The higher-order symmetries were explained using phenomenological symmetry-based arguments [5] due to (111) textured interface and Fuchs–Sondheimer theory for scattering at interfaces (see §2.3). All results were obtained at RT [148].

The symmetry of AMR in ultrathin Fe-monolayers on a GaAs interface changed depending on the number of monolayers. While for eight monolayers, a fourfold component was dominant, with decreasing number of monolayers to six and four, the fourfold component decreased. This was attributed to a change of symmetry due to transitioning from bulk-like to interface-like symmetry [149].

*The perovskite iron nitride Fe_4_N and the derived materials* CuFe_3_N [150] and Mn_4_N [34]. For the iron nitride case, we can distinguish in-plane AMR [121,151,152] and *transverse AMR* (magnetic field H rotated in the plane perpendicular to the current  j; comparable to AMR in the *ZX*-plane in figure 3 and not to be confused with *transversal AMR* or PHE) [36]. FeN_4_ in the matrix of Fe-doped GaN also exhibits AMR [106].

In all samples, a fourfold component of the AMR was found, for example in in-plane Fe_4_N below 30 K [121], and it is almost vanishing at higher temperatures. In *transverse* AMR of Fe_4_N and in Mn_4_N the fourfold component is dominant for low temperatures.

All samples show negative AMR at low temperatures. The AMR in Fe_4_N (in-plane) and in CuFe_3_N remain negative, while Fe_4_N (*transverse*) and Mn_4_N show positive AMR for temperatures above approximately 50 and 100 K, respectively. Low temperature AMR ratios for Fe_4_N and Mn_4_N scatter between approximately −0.75% [121] and −7% [152] in iron nitride and around 2% in manganese nitride [34]). While AMR ratios scatter in general, an increasing AMR ratio for increasing annealing temperature was reported in iron nitride [121]. In the ferromagnetic anti-perovskite *γ*′ − CuFe_3_N, low-temperature values in the range of −0.067 to −0.336% were reported, at higher temperatures dropping to 0.003%.

In iron and manganese nitride, the decrease of the AMR coefficients with increasing temperature show a kink at about 50 K, changing from rapid to moderate decrease. No explanation was given. The results were discussed in the framework of *sd*-models [27]. Negative (positive) AMR ratios were linked to minority (majority) spin conduction while appearing fourfold symmetries were linked to possible tetragonal distortion. In Fe_4_ this was suggested to be due to anisotropic thermal compression [36].

*Some more perovskites.* Metallic SrRuO_3_ exhibits negative MR [47] as expected for FMs, and its form for parallel and perpendicular configuration of magnetization and current confirms this is AMR rather than an orbital effect [153]. The AMR is negative and achieves quite large values of approximately 25% at low temperatures. It is slowly decreasing for low temperatures and steeply decreasing for higher temperatures above approximately 100 K as it is approaching and surpassing the Curie temperature of approximately 140 K. It does not show any enhancement in the vicinity of the Curie temperature, which is contrary to the results in manganites and was attributed to the absence of the Jahn–Teller effect [154]. However, the MR depends sensitively on strain [155]. AMR and PHE were compared at low temperatures and it was found that the AMR is almost double as large as the PHE with approximately 14% and approximately 7%, respectively [156], implying sizable crystalline AMR terms.

The non-magnetic SrIrO_3_ shows AMR for temperatures below 20 K, which was interpreted as a sign of a possible ferromagnetic ordering emerging at low temperatures induced by local structure distortion due to lattice strain. The presented data were close to a twofold AMR. More precisely, the fitting process yielded AMR ∝ cos (1.75*ϕ*) [157]. The relationship of this unusual result to magnetic order is discussed in that reference.

Interestingly, bulk SrIO_3_ does not show such behaviour and the here investigated film is a thin film on a SrTiO_3_ substrate [157]. In the previous section, we discussed various examples of thin films on STO substrates forming a 2DEG at about the same temperature accounting for the transport effects. In our judgement, this could account for the emergent AMR at low temperatures.

*Manganites* form a large class of perovskite materials ranging from the more common antiferromagnets such as CaMnO_3_ [158] to FMs (less common in ternary [159] and well-established in numerous quaternary systems described below). Often, this material class is defined as compounds of the form *X*_*a*_*Y*_*b*_MnO_3_, where X and Y are a trivalent and divalent cation, respectively, with their respective concentrations *a* and *b* [154]. The main part of manganites discussed here are based on lanthanum, for which the second element *Y* is either Ca [11,160–164], Pr [48,165], Sr [166] or Ag [167]. Among the other materials are Nd_0.51_Sr_0.49_MnO_3_ [168] and Sm_0.5_Ca_0.5_MnO_3_ [169]. In many of the studies a STO substrate was used [11,48,160,161,164,169], while sometimes also other substrates such as LAO [48] and BaTiO_3_ (BTO) [164] were reported. The role of the substrate in the results is here solely attributed to the strain it applies on the manganite layer. Lathanum-based oxides ordering in a perovskite structure on a STO substrate resemble at the first glance the LAO/STO samples discussed in the previous section in terms of the 2DEGs. The difference is that LAO is a non-magnetic insulator, where magnetism and transport are only occurring at the interface with the substrate.

The AMR is usually reported to be twofold; however, also fourfold AMR was reported. While the fourfold symmetry was reported to be robust in La_2/3_Ca_1/3_MnO_3_ (LCMO) [11], it only appeared on a tensile strained La_0.4_Sr_0.6_MnO_3_ sample on a STO substrate [166]. Other substrates showed twofold AMR for the same material. Arguably the most attention was paid to ferromagnetic LCMO where the colossal magnetoresistance (CMR) occurs, and here, the AMR at low temperatures [164] is clearly observable but small. At higher temperatures a peak was found in slightly off-stoichiometric LCMO, La_0.7_Ca_0.3_MnO_3_ films [161]. In the former case [164], it was ascribed to be due to strain from the BTO substrate.

The sign of the AMR is in most cases predominantly negative. However, some studies report a sign change of the AMR as a function of temperature [48,162,167,169], and others report exclusively negative results [160,165]. In the study of Xie *et al.* [164], a 80 nm thick sample on a BTO substrate was reported to show a sign change, while the other samples are solely negative. The sign change of the AMR was sometimes linked to a change of the easy axis with temperature.

**Figure 15.**
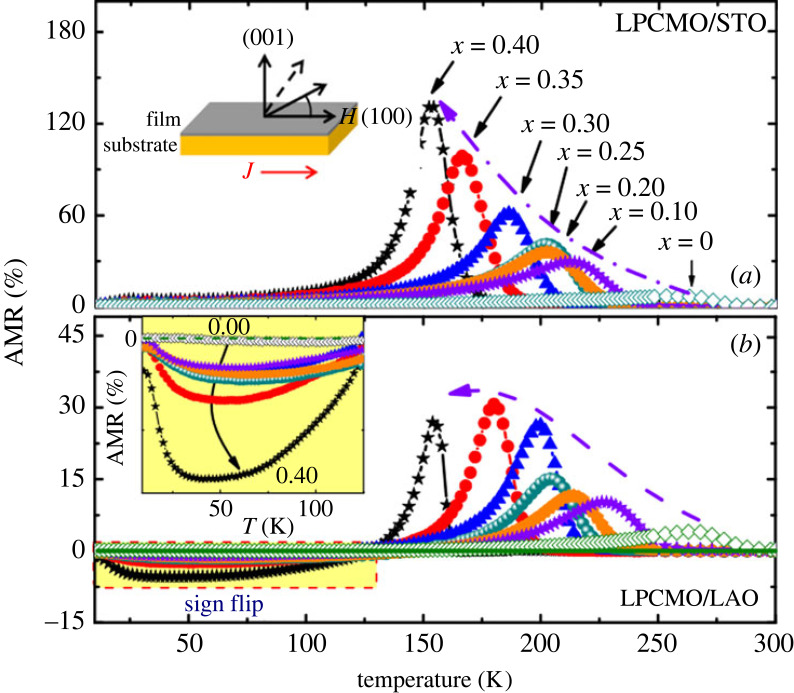
AMR in La_0.7−*x*_Pr_*x*_Ca_0.3_MnO_3_. Temperature dependence of AMR measured in a field of 1.1 T at doping levels *x* = 0, 0.10, 0.20, 0.25, 0.30, 0.35 and 0.40 for films grown on (*a*) SrTiO_3_ and (*b*) LaAlO_3_ substrates. Dashed curves in both figures show the expected dependence of the AMR_max_ on doping. The change of sign of the AMR with increasing doping is shown in the rectangular yellow area. Insets in (*a*) and (*b*) show the direction of magnetic field *H* and the direction of the current *J*, and the expanded view of the AMR at low temperatures for LPCMO/LAO-doped films, respectively. The figure is reproduced from Alagoz *et al.* [48].

Magnitudes of the AMR ratio are scattered between approximately 0.1% in LCMO [160] and ‘colossal’ values above 100% for La_0.3_Pr_0.4_Ca_0.3_MnO_3_ at its peak value approximately below 150 K [48] on a STO substrate as can be seen in figure 15*a*. AMR values were reported as highly sensitive to e.g. sample composition, type of substrates [48]—and thus strain—and current directions [160]. An example of the dependence of AMR on the substrate and the doping levels is shown in figure 15.

In an absolute majority of the studies, the low-temperature AMR increased with increasing temperature in clear contrast to the usual behaviour and peaked just below the metal–insulator transition temperature. Above this temperature, the AMR ratio decreased rapidly. The microscopic mechanism of AMR in these materials is distinct from conventional FMs (such as alloys of transition metals discussed previously) [170]. These transition temperatures are scattering from that of liquid helium [165] up to almost RT [162]; however, usually somewhat lower than the latter. Exceptions to the high-temperature peak are rare and occur for example in thin-film samples of LCMO on a BTO substrate [164], in La_0.4_Sr_0.6_MnO_3_ [166] and in a polycrystalline Nd_0.51_Sr_0.49_MnO_3_ sample [168], while the single-crystalline samples of the latter show the characteristic peak.

The explanation for the characteristic behaviour is usually linked to strain resulting in orbital deformation via the Jahn–Teller effect [48,161,166,167,169]. Other authors offered explanations linked to double exchange [164] or to a magnetic liquid behaviour [165].

It is worth noting that the ‘colossal’ peak values of AMR can be usually observed in the vicinity of the MIT-temperature, where also the CMR effect occurs. Thus, explanations of these large AMR values have to be taken with some caution as neither the CMR effect is fully understood. In the latter case mainly due to a lack of quantitative theory describing the MIT and the subsequent insulating phase, which in our judgement might be problematic in the description of the AMR as well understood. In contrast, MIT in EuTe_2_ is well understood (see §3.3).

*Other conductive oxides.* In Fe_3_O_4_ (magnetite), AMR was used to refute predicted half-metallicity [47], and later, a strong crystalline contribution was reported [171]. The origin of twofold and fourfold AMR in magnetite was linked to magnetic anisotropy and to scattering far away and near the antiphase boundaries, respectively. This oxide can be alloyed with nickel while remaining conductive: AMR in Ni_0.3_Fe_2.7_O_4_ shows a strong fourfold component as well [171]. Another example of a conducting oxide is the AFM RuO_2_. Here, angle-dependent spin-torque ferromagnetic resonance measurements (ST-FMR) in fig. 3b,d of [172] resemble AMR and indicate the existence of the effect in the material.

*TMDC-based magnetic compounds.* Being non-magnetic themselves, some transition-metal dichalcogenides (TMDCs) allow for insertion of magnetic atoms as reviewed in the introduction of [173] and ferromagnetic or antiferromagnetic order may arise.

In Mn_1/4_NbS_2_, thus Mn-inserted NbS_2_, ferromagnetism occurs below its Curie temperature of *T*_*C*_ = 104 K, while for higher temperatures it is paramagnetic. Furthermore, first-principle calculations suggest a pressure-induced transition to a AFM state. Experiments were conducted at various temperatures for fields up to 9 T. The comparison LMR and TMR measurements allow to conclude that there is no AMR in the paramagnetic state, while differences in LMR and TMR in the FM state for large enough fields show AMR of up to approximately 9% at 9 T and *T* = 2 K [173].

Fe_0.28_TaS_2_ is ferromagnetic below *T*_*C*_ = 68.8 K. Angle-dependent MR measurements confirm the existence of AMR, which is slightly larger than 10% for the saturated state at *T* = 10 K for *B* = 8 T. MR of this compound is about 100 times larger than for the similar Fe_0.25_TaS_2_ [174]. Similar results were found in single-crystalline Mn_1/3_TaS_2_. Next to FM order (*T*_*c*_ ≈ 70 K), measurements indicated the existence of cluster spin glass at low temperatures. AMR, which can be calculated by comparing LMR and TMR, is in a similar order of magnitude as for the previous compound of Fe_0.28_TaS_2_ [175].

Contrasting the simpler types of magnetic order of the previously mentioned materials, Cr_1/3_NbS_2_ is identified as a chiral helimagnet below the critical temperature *T*_*C*_ = 111 K. Both AMR and PHE show a twofold behaviour of less than 1% magnitude [176]. Lastly, in V_5_S_8_, magnetization studies suggest an antiferromagnetic ordering around *T*_*N*_ ≈ 27 K. Low-temperatures AMR measurements show a twofold AMR of up to 1% magnitude [177].

## 4. Applications and further topics

There is a broad range of opportunities to exploit the AMR and also to go beyond magnetization-controlled DC resistance. In the following, we will shortly discuss both industrial and scientific applications of this effect, ranging from the well-known AMR sensors used in a variety of fields to subtle techniques for the detection of spin relaxation in FMs. Related phenomena in optics and thermoelectricity will be mentioned as well.

### 4.1. Scientific applications

The difference between *scientific applications* discussed here and the results of numerous AMR experiments in the previous section is the direction of reasoning: while the measured AMR values and their symmetry were used to reach conclusions about the inner workings of the AMR, now the AMR as an effect is known and so the measured values are used to detect another quantity, mostly the magnetization direction.

Since AMR gives the dependence of the resistivity on the magnetization direction, it can be used as a means of *magnetometry*. This is the main application of AMR in a scientific context and a few examples are given in the following paragraph. This section also summarizes a variety of other applications of AMR measurements and theory.

A new ferromagnetic resonance (FMR) method using AMR was developed by Fang *et al.* [178]. There, an electrical current at microwave frequencies is used to induce an effective magnetic field in nanoscale bars of (Ga,Mn)As and (Ga,Mn)(As,P), which are then probed by voltage measurements and analysed within the framework of non-crystalline AMR [178]. Comparable techniques were employed to detect RT spin–orbit torques [179] in the half-Heusler compound NiMnSb [129] and RT spin-transfer torques in a structure consisting of the topological insulator Bi_2_Se_3_ and permalloy [180].

In another context, the AC susceptibility of thin films of Co, Ni and nickel alloys was determined by voltage measurements. The expression for the susceptibility (eq. 6 in [181]) was derived using the non-crystalline AMR [181].

And lastly, magnetization reversal was studied by AMR (amongst other means) in nickel nanowires [182]. However, here the term AMR refers to resistance measurements being subject to magnetic field sweeps at different field directions, similar to aforementioned publications which list longitudinal and transversal MR separately. Jumps in the resistance signal are taken as an indication of pinning and unpinning of the magnetic domain walls in the magnetization reversal process. Comparable works can be found in [50,183,184]. A similar study, however, with a focus on detecting and characterizing the domain wall itself, can be found in [185].

A frequently invoked concept of the AMR theory is the *sd*-scattering, which has been mentioned in many positions in this work already. Usually, theoretical predictions of the strength of *sd*-scattering lead to predictions about the AMR. The opposite is, however, also possible: a very small non-crystalline AMR of 0.001% was used to argue that *sd*-scattering is repressed, the electron carriers and Fermi level reside in the conduction band and the main scattering process is s-s-scattering [63,186].

While the *sd*-scattering is governing AMR, its reverse process the *d* → *s* electron scattering is involved in *spin relaxation*. A spin-relaxation theory suitable for nickel- and cobalt-based alloys based on the theory of AMR of Campbell, Fert and Jaoul [16,187] was developed by Berger and exemplified on permalloy. Parameters of the model were deduced from existing AMR data [188].

The angular dependence of AMR was used in various occasions: first, in quantifying the current-induced Rashba fields in LAO/STO heterostructures and investigating their dependence on applied magnetic field and on electric field modulation [189]. The LAO/STO heterostructures forming a 2DEG, which is more extensively discussed in §3.5.

Secondly, the signals of inverse spin Hall effect (ISHE) and AMR are typically mixed and thus knowledge about AMR is crucial to quantify the spin-Hall angle correctly. In [190,191] methods show how to disentangle their signals by symmetry. The ISHE was investigated in permalloy/Pt bilayers [190] and Pt, Au and Mo [191], respectively. AMR can be used to probe the dimensionality of the FS as was for example done for Ca_0.73_La_0.27_FeAs_2_ single crystals, where it was argued that the FS is quasi-two-dimensional [192].

### 4.2. Unconventional examples and related effects

This section attempts to give a short overview of AMR-related research outside the mainstream, such as the investigation of AMR in non-collinear systems (see §4.2.3) where no single spin direction can be defined as in FMs (net magnetization) or collinear antiferromagnets (Néel vector); as well as discussion of similar effects which can partly make use of AMR terminology as such its thermoelectrical counterpart, the AMTP discussed in §4.2.2 below.

#### 4.2.1. Frequency-dependent anisotropic magnetoresistance

This review focuses on AMR in the DC regime. Conductivity is, nevertheless, a function of frequency *σ*(*ω*) and so is its anisotropy. It is meaningful to divide the following discussion into low and higher frequencies. Given the typical scattering rates 1/*τ* in electrically conducting materials, the former means terahertz while the latter spans the visible range and beyond. In the following paragraph, we discuss AMR in the terahertz regime.

The special aspect of the terahertz range is that *σ*(*ω*) is dominated by intraband contributions which are usually well approximated by the Drude peak, *σ*(*ω*) ∝ (1 − *iωτ*)^−1^, where *τ* is the transport relaxation time. It is then possible to split [15,193] AMR into

4.1 $$\mathrm{AMR}=\frac{\sigma_{⊥}-\sigma_{∥}}{\sigma_{⊥}}=\frac{A}{1-i\omega \tau }+B.$$

Since the *ω*-independent term *B* happens to be a function of the intrinsic AMR and the *ω*-dependent part of the extrinsic AMR, the ac-AMR offers a possibility for experimentally distinguish these two quantities (see §§1.1 and 3.1) [15]. In this fashion, Co, Ni, Ni_50_Fe_50_ [15] and permalloy [15,193] were investigated (see §3.1 for the discussion). Please note that the frequency-dependence was not investigated by means of AC measurements, but the samples were instead subjected to an incident polarized electrical pulse in THz frequency. After transmitting through the sample, the outgoing pulse was detected [15,193].

Beyond the THz range, interband terms become important, see eq. B6 in [194]. At these higher frequencies (*ωτ* ≫ 1), the focus turns to magneto-optical effects which are even in magnetization, such as the Voigt effect or its analogy in reflection (see fig. 2 in that reference for an overview) as counterparts to AMR in the DC-limit. Spectral measurements then provide information about the valence band structure: iron [195], (Ga,Mn)As [194] or Heusler compounds [196]. Going even further in frequencies, X-ray magnetic linear dichroism (XMLD) [197] involves physics of atomic core levels, but these effects go beyond the scope of this review.

#### 4.2.2. Anisotropic magnetothermopower

The AMTP is the thermoelectric counterpart of the AMR. Among linear response coefficients

$$j=eL_{11}E+L_{12}\nabla T$$

and

$$j_{Q}=eL_{21}E+L_{22}\nabla T,$$

it is not only *L*_11_ = *σ*/*e* that may depend on magnetization direction [198]. Off-diagonal terms of the *L*_12_ tensor correspond to the anomalous Nernst effect (named in analogy to the AHE manifested in off-diagonal terms of *L*_11_), and the AMR (in *L*_11_) has the AMTP as its counterpart in *L*_12_. Magnetoanisotropy of all these coefficients can be anticipated [199]; they are tensors bound by Onsager relations (see equation 1.6) [200].

Literature is sparse since measurements and calculations are both challenging. The measurements of *L*_12_ are challenging due to possible unwanted thermoelectric contributions which hardly can be averaged out [12]. In the case of the calculations, the challenge lies in properly evaluating the derivatives, $L_{12}∼\int v_{k}^{2}\delta^{\prime }(E_{k}-E_{F})$. In the following, we provide a few examples on AMTP.

Quantitative studies based on phenomenological symmetry-based models analogous to the approach presented in §2.1 are conducted in Co_2_MnGa [12] and in (Ga,Mn)As [9]. While in Co_2_MnGa only crystalline and non-crystalline AMTP components up to the second order were confirmed, higher-order components have been identified in (Ga,Mn)As. In both cases, AMR and AMTP components were not directly related. More examples of AMTP studies in (Ga,Mn)As can be found in sec. III-D-2 of the review by Jungwirth *et al.* [201].

Concerning the *L*_22_ coefficient, the AMR was compared by Kimling *et al.* with *anisotropic magnetothermal resistance effect (AMTR)* in polycrystalline Ni nanowires [89] for a range of temperatures. The AMR and AMTR are expressed as ratios and the AMTR is found to be weaker than the AMR due to electron–magnon-scattering. A two-current model for AMTP in analogy to the work of Campbell, Fert, and Jaoul on AMR [16,187] was derived by Heikkilä *et al.* [202].

#### 4.2.3. Non-collinear systems

For a long time, the AMR was only associated with FMs. However, the discovery of AMR in collinear antiferromagnets, as described in §3.3, demonstrated that AMR can also occur in other magnetically ordered materials. Collinear AFM can have only two MSLs (typically denoted as *spin up* and *spin down*) which are aligned parallel to each other and allow for the definition of a SSA (the Néel vector); practical differences between these two cases (compare the SW1 and SW2 models discussed in §1.5) are small nevertheless. Collinear AFMs alone, however, do not exploit the set of zero-net-magnetization systems to the fullest. In the case of non-collinear antiferromagnets, all magnetic moments do point in the same plane; however, it is not possible to define a SSA as the Néel vector. An example is magnetic ordering on a Kagome lattice in Mn_3_Sn [57] or on a trigonal lattice in CrSe [203]. In the latter case, magnetic moments do not lie in the same plane and such non-coplanar magnetic order can bring about unexpected consequences. As such, both non-collinear and non-coplanar antiferromagnets pose a challenge to the classical definition of the AMR (equation (1.1)), as we discuss below. On the other hand, this class of systems also offers AMR mechanisms unavailable for collinear magnetic order.

**Figure 16.**
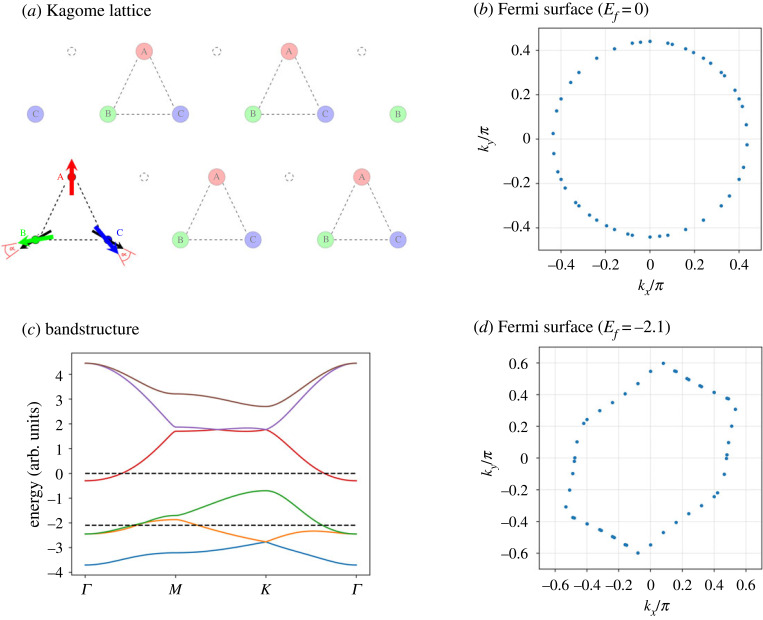
Archetypal non-collinear system: Kagome lattice with three MSLs. (*a*) Configuration of magnetic moments parametrized by tilting angle *α*. (*b*,*d*) Examples of Fermi surfaces with *α* = 0° and 10°, respectively. (*c*) Band structure with Fermi level indicated.

Ever since the work of McGuire & Potter [1], it has been generally accepted that AMR is an effect relying on the SOI. This is, however, only true in collinearly ordered structures. A non-collinear or non-coplanar order can mimic some properties of the SOI as, for example, it was shown for AHE on a distorted fcc lattice endowed with non-coplanar magnetic order [204]. Non-collinear order is sufficient to generate an anisotropic FS, which is thus causing intrinsic AMR as we demonstrate in figure 16: an s-d model on Kagome lattice [205] yields an isotropic FS as evidenced in panel (*b*) for a symmetric ($\sum m_{i}=0$) configuration of magnetic moments. Figure 16*d* shows that for certain lower-symmetry configurations of the magnetic moments with $\sum m_{i}\neq 0$ the FS becomes anisotropic even in the absence of SOI. We stress that the hexagonal warping alone (when appreciable) does not break the isotropy in the sense of *σ*_*xx*_ = *σ*_*yy*_ as discussed in §2.3, but the additional oblique distortion does. Note that anisotropies in scattering could result in additional extrinsic AMR, and additional intrinsic AMR could arise when SOI is accounted for.

Broadly speaking, AMR means that the electric resistivity (observable) is influenced by a well-defined parameter. For non-collinear magnets, the cleanest way of defining such anisotropy is by assuming that this parameter is an external magnetic field (that can be rotated, for example), which is also the most practical from the point of view of an experiment [206,207]. This approach, however, ignores the alternative means to manipulate magnetic moments: spin–orbit torques (both in FMs and antiferromagnets [179]) or piezo-stressor control of magnetic anisotropy [208] to name just two examples. Focusing on magnetic field in the context of AMR, however, we first consider it does not directly alter electric resistivity and only the direction of magnetic moments (whereas their change produces the change of resistivity). The fact that resistivity can be directly influenced by orbital effects is disregarded in this approach and thus, analysis of measured data can become complicated. Therefore, both definitions are practically acceptable: first, for reasons of practicality to directly jump from magnetic field rotation to the resistivity, and second, to consider how the direction of the magnetic moments influences the resistivity.

#### 4.2.4. Exotic phenomena

In the context of anisotropic magnetotransport, research focuses mostly on the diffusive regime in bulk systems. In the following, we wish to mention several phenomena outside this realm. First, AMR in the ballistic transport regime is discussed, followed by a quick look at TAMR and AMR in topological insulators.

*Transport in the diffusive regime* is dominated by scattering, described by the mean-free path of the carrier. When the sample size becomes smaller than the mean-free path we are talking of the ballistic regime: carriers are only scattered at the boundaries of the sample and can otherwise travel unhindered. Ballistic transport is often related to one-dimensional structures such as nanowires. It is possible to have AMR in this regime, which is subsequently either called *ballistic AMR* [209] (BAMR) or *quantized AMR* (QAMR) because of its stepwise character [210]. The BAMR is an effect similar to intrinsic AMR, since in both cases no external scattering is responsible for the effect. In the case of BAMR, the number of bands at the Fermi level and thus the ballistic transport changes with the magnetization direction [209]. It was found that the BAMR is a step function with the magnetization angle [209,210]. The step-like behaviour is only found at low temperatures and for small sample sizes. Increasing the size changes the number of conduction channels and leads to smearing out of the step. An increase of temperature likewise smears out the step [210]. In the latter cases, nickel [209] and iron [209,210] have been investigated. This list of phenomena going beyond traditional AMR is by no means complete and we refer the reader to the review by Zhao *et al.* [211] at this point.

The dependence of ballistic AMR on an ideal infinite monoatomic iron wire was compared with influences of domain walls and contacts, both of which can alter the transport properties significantly [212]. And lastly, AMR in a Rashba 2DEG was compared between the diffusive and the ballistic regime. The diffusive AMR can be large at low carrier densities which was attributed to the dependence of density of states, while the ballistic AMR shows a nonlinear dependence on the exchange, which was attributed to FS effects [14].

*Tunnelling AMR* can be understood as a crossover of AMR, where the anisotropy, thus changing of resistivity with magnetization direction, is important; and the tunnelling magnetoresistance (TMR) [213,214], which is based on tunnelling as encrypted in its name. Seminal work by Gould *et al.* [215] carried out on a structure of a ferromagnetic (Ga,Mn)As layer, a tunnelling barrier and a non-magnetic material attributed the TAMR to the anisotropy of the partial DOS [215]. Recently, Schöneberg *et al.* [216], found that Pb dimers on a ferromagnetic surface show different STM currents depending on the crystalline orientation. For a [001]-oriented dimer, the TAMR reached up to 20%, linked to a difference of the local DOS (LDOS) depending on the magnetization direction, while TAMR is absent for a [111]-orientation due to only a small difference of LDOS depending on magnetization [216]. The TAMR is very much a topic of its own, only related by analogy to the original AMR effect, and further discussion is beyond the scope of this review.

Kandala *et al.* [217] found that in the Cr-doped topological insulator (Bi,Sb)_2_Te_3_, which is a FM up to 8 K, exhibits a giant AMR of more than 120% in a rotation from out-of-plane to in-plane. This is because, for an out-of-plane field, a magnetic gap opens for the surface states (quantum AHE), but when the field is in-plane, surface states are restored [217].

Beyond charge transport, an analogy of AMR in transport of magnons in an ferromagnetic insulator has been observed. Measurements of *magnon AMR (MAMR)* and a *magnon PHE* were reported [218] in a series of films of either 100 or 200 nm thickness. The MAMR and MPHE were defined analogous to the classical equation (1.1), where classical resistivities are replaced by the average magnon conductivity parallel or perpendicular to the magnetization. The magnon conductivity is defined in an analogous fashion to the classical conductivity as current of magnons per unity temperature gradient. The magnons are measured indirectly as per the first and second harmonic of the AC current. More details to the experimental realization can be found in [218]. The MAMR and MPHE is of approximately 5% magnitude.

Moreover, in various cases, a *proximity-induced AMR* was measured in bilayers consisting of a non-magnetic conductor and a magnetic insulator. As conductors served Pt [219], Pd [220] and the topological insulator Bi_2_Se_3_ [221,222]. The magnetic insulator was yttrium iron garnet (YIG) and in one case also thulium iron garnet (TmIG) [222]. The angle-dependence of the proximity-induced AMR was generally twofold [219,220,222], only in Pt at very low temperatures the angle-resolved AMR showed a more complex signal due to overlap with spin Hall magnetoresistance (SMR). In the Pt bilayers, the experiment was conducted in three different rotation planes, analogous to Ritzinger *et al.* [12]. The AMR has a maximum value of approximately 0.3%, generally smaller for out-of-plane AMR than for the in-plane AMR [219]. Also for the Pd bilayers, a coexistence of AMR and SMR were noted. AMR was vanishingly small with approximately 0.012% at low temperatures and even smaller for higher temperatures [220].

In the Bi_2_Se_3_-bilayers, the magnetic field was rotated in an out-of-plane rotation plane. Positive AMR ($R_{∥}>R_{⊥}$) was found. The proximity effect was confirmed, since in bilayers of Bi_2_Se_3_ and Al_2_O_3_ no AMR signal was found [222]. The magnetotransport phenomena in Bi_2_Se_3_ were attributed to surface-state contribution rather than to bulk conduction [221,222]. LMR and TMR data suggest that part of the angular signal originates from magnetism, while other parts stem from orbital effects [222].

In all these cases, proximity-induced magnetism has led to the occurence of AMR. We conclude by the observation that AMR in bilayers such as Fe on GaAs, already discussed in §3.6, is a similar effect where, however, magnetism of the conducting layer is only modified (not introduced) by the proximity of a material with different symmetry.

### 4.3. Industrial applications

To date, the AMR effect is widely exploited in sensors as a direction-sensitive magnetometry probe to obtain quantities such as absolute position or rotation speed [223–225].

Important is the usage in the automotive industry [226], in biomedical applications [227–230] and in aerospace missions [231–234]. Examples in the automotive industry include sensing of crank shaft position, wheel and transmission speed, and throttle valve position for air intake [226]. A list of further applications can be found at the beginning of [226]. For biomedical applications, AMR sensors are used as biosensors for detecting magnetically labelled targets [228–230], which have certain advantages over e.g. fluorescent-based labelling, such as greater sensitivity, longer time stability, being more remotely controllable and showing less noise [229]. In aerospace missions, AMR sensors are used as magnetometers for scientific purposes and detection of the absolute position of the spacecraft. Here, the ability to detect Earth’s magnetic field with sufficient precision is needed [231–234].

Further examples outside of these fields include weak field measurements [225], such as in a compass [235], traffic detection and measurements of current [225,236]. The measurements of current are taking advantage of Ampere’s Law where the AMR sensors detect the magnetic field induced by a current flowing through a wire [236]. Please note that sensors based on giant magnetoresistance (GMR) and tunneling MR are often mentioned alongside AMR [227,229,232]. AMR sensors offer quite a few advantages, which explains their popularity in applications. They can be [224,226] produced at low cost [227,229,231,237], are quite small [224,227], achieve a high sensitivity [224,225,233,234] with resolution well below millimetre or degree range and are still working if there is a gap between sensor and magnet [224], to name only a few. In comparison with Hall sensors they are superior in terms of sensitivity [236], lower cost and endurance to mechanical stress [226]. Especially appreciable for biomedical application is that AMR sensors offer a remote, thus non-invasive, way of detection [228,229]. Requirements for materials used in AMR sensors include large AMR signal (high signal to operating voltage ratio), large *ρ*_0_ (associated with low noise [225,227,234]), low anisotropy, low sensitivity to magnetostriction, long-term stability [225]. Wide temperature ranges are required for operation especially in automotive and aerospace applications as the temperature can vary by more than $100^{\circ }C$. AMR sensors many times offer a suitable (linear) temperature dependence [226,229,233], which can be accounted for electronically [226]. Commonly used materials are mainly basic transition metals discussed in §3.1 and especially permalloy [225,227–230,234,237,238]. The latter has many of the desired properties.

In application-based publications, anisotropic MR is considered as non-crystalline AMR [224,225,237] only, crystalline components are not relevant because of polycrystallinity of used materials. The noise in AMR sensors is typically dominated by magnetic fluctuations [223]. Please note that similar to AMR sensors, the transverse PHE can be used to fabricate PHE sensors [238].

Arguably the best-known applications of AMR fall into the realm of magnetic memories. Early magnetoresistive random access memories (MRAM) were based on the effect, yet it is much smaller in magnitude than GMR which eventually prevailed [239]. Modern MRAMs are based on the tunneling MR effect [240]. To date, AMR is still used in applications related to the conventional hard drives [240] where information is read using a multilayer device [223,237] whose resistance changes depending on the magnetic state of the free layer. AMR must also be considered [241] in the design of racetrack memories. In the recent decade, numerous attempts of developing novel spintronic applications based on antiferromagnets (see §3.3) were made. In the proposed applications, AMR and its transversal counterpart the PHE were considered as a readout mechanism [51,114,117]. To date, while spintronic applications have been numerous, none of them contains active AFM elements, so in this context, AMR is only exploited in FMs.

## 5. Conclusion

Magnetotransport in solids is a vast and mature field. In this review, we focus only on a small part of it, namely its anisotropy related to magnetic order. AMR usually refers to this phenomenon, albeit occasionally orbital effects are also included (and these are not covered in this review). Two characteristic features of magnetism are helpful to this end: remanence and coercivity. Unlike ordinary MR which just happens to be anisotropic, the AMR can usually be observed as a spontaneous effect even at zero field; on the other hand, well above coercive field, MR traces should run in parallel regardless of the experimental configuration (e.g. magnetic field parallel and perpendicular to current). Microscopically, the AMR can either originate from anisotropic scattering or band structure deformation (related to magnetic order) which is analogous to the extrinsic and intrinsic mechanism of the AHE. This analogy is not very deep, however, as it can be exemplified with the intrinsic AMR which is unrelated to Berry curvature of Bloch states.

The phenomenological understanding of the AMR is based on symmetry analysis of the resistivity tensor, and the basic distinction of non-crystalline (equation (1.2)) and crystalline (or mixed) terms allow to distinguish single crystals from polycrystals where only the former occurs. Models as in equation (2.2) offer a workable description of higher-order crystalline AMR in any crystal symmetry. Absolute and relative values of the AMR coefficient in equation (1.2), as a material parameter, are useful for polycrystals and single crystals, respectively. While the latter is usually used, one should be careful: sputtered films of the same material will exhibit different relative AMR depending on the strength of scattering on grain boundaries (which is typically unrelated to magnetism). Strong variations of published AMR values are, therefore, to be expected.

AMR has been investigated in a vast range of materials, starting with the simple and elemental ferromagnets Co, Fe and Ni and their alloys, of which especially permalloy has received great attention due to its favourable properties for applications. Other materials in which significant investigations have been carried out include the dilute magnetic semiconductor (Ga,Mn)As, antiferromagnets as CuMnAs and MnTe, Co-based Heusler alloys, two-dimensional electron gases on LAO/STO interfaces, manganites and other perovskites. Many more materials have received at least a little attention. The magnitude, sign, symmetry and inner mechanisms of AMR in these vastly different material classes vary heavily.

While the AHE has attracted considerably more attention than AMR in fundamental research, the situation is quite the opposite in commercial applications. Contrary to AHE, the AMR has already made it to the market-ready stage in the niche of various sensors still widely used to date (as traffic detection, biomedical and aerospace applications, and more) and also in the past in magnetic memories. Scientific applications of AMR, such as a means to determine magnetization direction in situations where other methods fail, have also become important. More work is needed, however, to close the gap between real-world applications and the large body of fundamental research that has been carried out on AMR over the last 165 years.

## Data Availability

Electronic supplementary material is available online [243].

## Footnotes

1 Intrinsic anomalous Hall effect is one of the exceptions: off-diagonal conductivity component can be expressed in terms of an integral of so-called Berry curvature over Fermi sea (rather than Fermi surface). For DMS, this has been done for example in [65].
